# Colloidal crystal engineering with metal–organic framework nanoparticles and DNA

**DOI:** 10.1038/s41467-020-16339-w

**Published:** 2020-05-19

**Authors:** Shunzhi Wang, Sarah S. Park, Cassandra T. Buru, Haixin Lin, Peng-Cheng Chen, Eric W. Roth, Omar K. Farha, Chad A. Mirkin

**Affiliations:** 1 Department of Chemistry, Northwestern University, 2145 Sheridan Road, Evanston, IL 60208 USA; 2 International Institute for Nanotechnology, 2145 Sheridan Road, Evanston, IL 60208 USA; 3 Department of Materials Science and Engineering, Northwestern University, 2220 Campus Drive, Evanston, IL 60208 USA

**Keywords:** Metal-organic frameworks, Organic-inorganic nanostructures, Organizing materials with DNA

## Abstract

Colloidal crystal engineering with nucleic acid-modified nanoparticles is a powerful way for preparing 3D superlattices, which may be useful in many areas, including catalysis, sensing, and photonics. To date, the building blocks studied have been primarily based upon metals, metal oxides, chalcogenide semiconductors, and proteins. Here, we show that metal–organic framework nanoparticles (MOF NPs) densely functionalized with oligonucleotides can be programmed to crystallize into a diverse set of superlattices with well-defined crystal symmetries and compositions. Electron microscopy and small-angle X-ray scattering characterization confirm the formation of single-component MOF superlattices, binary MOF–Au single crystals, and two-dimensional MOF nanorod assemblies. Importantly, DNA-modified porphyrinic MOF nanorods (PCN-222) were assembled into 2D superlattices and found to be catalytically active for the photooxidation of 2-chloroethyl ethyl sulfide (CEES, a chemical warfare simulant of mustard gas). Taken together, these new materials and methods provide access to colloidal crystals that incorporate particles with the well-established designer properties of MOFs and, therefore, increase the scope of possibilities for colloidal crystal engineering with DNA.

## Abstract (web summary)

Colloidal crystals assembled from nanoscale building blocks are powerful designer materials with diverse functionalities. Here, the authors describe a colloidal crystal engineering strategy to prepare hierarchical structures from metal–organic framework nanoparticles and DNA which retain permanent porosity and catalytic activity.

## Introduction

Colloidal crystal engineering with nanoparticles (NPs) has emerged as a powerful tool to design materials from the bottom up^1–4^. When NP building blocks are combined with nucleic acids, they may behave as programmable atom equivalents (PAEs) and can be assembled in a sequence-specific fashion into crystalline arrangements driven by a combination of DNA complementarity and their unique nanoscale architectural features (e.g., dimensions and anisotropic shapes)^5–11^. Early studies focused primarily on particles composed of gold NP (AuNP) cores, due to the extensive methodology available for synthesizing them in monodisperse form under aqueous conditions; such particles can be modified easily with dense layers of alkylthiol-functionalized DNA^12,13^. Later, studies expanded the pool of available particles to a variety of PAEs, including other metals, metal oxides, chalcogenide semiconductors, polymers, and proteins^14–17^. A conclusion derived from these studies is that the resulting colloidal crystal quality is highly dependent upon PAE monodispersity^18–20^. Indeed, the almost perfectly monodisperse protein particles yield structures with well-defined crystal symmetries, highly tunable lattice parameters, and extremely well-formed crystal habits, all corroborated by electron microscopy and small-angle X-ray scattering (SAXS) measurements^16^.

Metal–organic frameworks (MOFs) are a class of highly modular materials with well-defined three-dimensional (3D) architectures, permanent porosity, and diverse chemical functionalities, and are promising for a wide variety of applications, including gas storage and separations^21,22^, drug delivery^23^, chemical sensing^24,25^, and catalysis^26^. NP forms of MOFs have similar properties but are dispersible in solution and therefore could become the basis for building blocks used in colloidal crystal engineering^27,28^. However, the polydispersity and poor colloidal stability of MOF NPs thus far limit their potential in this regard and therefore the types of colloidal crystals that can be engineered^29^.

**Fig. 1:**
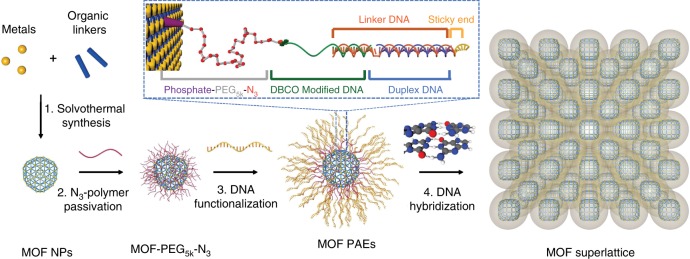
Preparation of MOF PAEs as building blocks for colloidal crystal engineering. Metal ions and organic ligands were first combined to synthesize MOF NPs. The MOF NPs were surface passivated by a layer of azide-modified polyethylene glycol (PEG) polymers, followed by DNA functionalization via copper-free click chemistry, and subsequently programmed into superlattices via DNA hybridization. Inset: heterobifunctional ligands and DNA strands used to engineer MOF NP superlattices consist of: (i) a phosphate-PEG_5k_-azide ligand that strongly coordinates to the MOF surface SBU, (ii) a diarylcyclooctyne (DBCO) moiety with an 18 base recognition sequence that binds to a DNA linker, (iii) a linker hybridized with a complementary sequence of desired and programmable length to control interparticle distances, and (iv) a “sticky end” sequence that drives sequence-specific MOF PAE assembly.

Herein we describe a density gradient centrifugation-based method for obtaining monodisperse samples of MOF NPs, employ a straightforward coordinative strategy for chemically modifying them with DNA at a density sufficient to support their programmed crystallization, and then explore how they can be assembled deliberately into superlattices based upon insight from the complementary contact model (Fig. 1)^8^. Significantly, the use of MOFs in colloidal crystal engineering allows one to utilize the unusual shapes that define MOF NPs as a structure-influencing factor^27,30–32^. Taken together, these studies provide access to a new set of colloidal crystals that incorporate particles with the designer properties of MOFs and therefore may dramatically increase their scope of utility.

## Results

### Synthesis of uniform and colloidally stable MOF PAEs

**Fig. 2:**
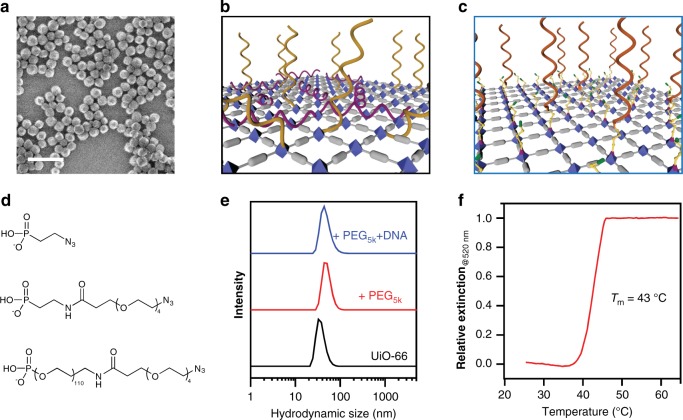
Synthesis and characterization of UiO-66 MOF PAEs. **a** SEM image of UiO-66 MOF NPs. Scale bar is 100 nm. **b** Schematic illustration showing how direct DNA surface functionalization suffers from multi-valent DNA backbone binding to MOF surface SBUs. **c** The proposed strategy passivates the MOF surface with an azide-functionalized PEG polymer layer that (1) allows alkyne-modified DNA to be effectively coupled, (2) prevents DNA backbone binding, and (3) increases MOF NP colloidal stability. **d** Three phosphate/phosphonate-PEG-azide ligands with varying number of ethylene glycol units were studied. **e** DLS analysis of as-synthesized UiO-66 NPs in water (black), PEG_5k_-modified UiO-66 NPs (red), and DNA functionalized UiO-66 PAEs in 0.5 M NaCl (blue). **f** Melting transition of MOF–Au PAE DNA-linked aggregates, monitored as a function of change in extinction at 520 nm via UV-vis spectroscopy.

As a proof-of-concept, Zr_6_O_4_(OH)_4_(BDC)_6_ (UiO-66, BDC = 1,4-benzenedicarboxylate) MOF NPs, composed of Zr_6_O_4_(OH)_4_ clusters (inorganic secondary building units (SBUs)) and terephthalic acid organic ligands, were modified with DNA and studied as PAEs. In a typical experiment, spherical UiO-66 NPs (diameter = 37 ± 8 nm) were synthesized via an acetic acid modulated solvothermal reaction based on minor modifications of literature methods (see Supplementary Information)^33^. To increase particle monodispersity, a post-synthetic density gradient centrifugation method was employed to remove particles from both the large and small end of the distribution (see Supplementary Methods), yielding UiO-66 NPs with increased size uniformity (37 ± 4 nm, Fig. 2a).

Unlike the surfaces of conventional inorganic NPs, which are typically capped with organic ligands, a mixture of positively charged metals and negatively charged ligands are exposed at the surfaces of as-synthesized MOF NPs^34^. Therefore, DNA surface functionalization, either through ligand post-synthetic modification or SBU coordination, inevitably suffers from competition with DNA itself as a ligand (Fig. 2b)^35–37^. To address this issue, a series of heterobifunctional polyethylene glycol (phosphate/phosphonate-PEG_*n*_-N_3_) ligands were used to modify the MOF NP surface prior to DNA functionalization. This approach passivates the MOF surface with respect to unmodified DNA but still allows alkyne-modified DNA to effectively couple to the MOF surface (Fig. 2c)^38^.

To determine the optimal PEG chain length, phosphate/phosphonate-PEG_*n*_-N_3_ surface-capping ligands with three different PEG chain lengths (from top to bottom: ethylene, PEG_4_, and PEG_5k_) were synthesized and studied (Fig. 2d). In a typical experiment, the PEG ligands were reacted with MOF NPs in dimethylformamide for 2 days at room temperature. ^31^P magic angle spinning solid state nuclear magnetic resonance spectroscopy confirmed that the phosphate is coordinated to the Zr^4+^, as evidenced by a characteristic 4.2 ppm up-field shift of the single phosphorous resonance upon surface functionalization (see Supplementary Figs. 12 and 13)^39,40^. Consistent with this conclusion, dynamic light scattering showed that UiO-66 NPs increased in average size from 37 ± 8 to 48 ± 12 nm (polydispersity index = 0.05) upon surface modification with PEG_5K_ (Fig. 2e). Importantly, of the three PEG ligands studied, the PEG_5k_ structure was most effective at stabilizing the MOF colloid and making the particles suitable for further functionalization with DNA. Indeed, significant particle aggregation in 0.5 M NaCl solution occurred with the shorter ligands (See Supplementary Fig. 10). The PEG_5k_ surface coverage was determined to be ~2 molecules/nm^2^.

To prepare the PAEs, the azide-terminated MOF NPs were reacted with terminal diarylcyclooctyne-modified DNA (5’ DBCO-TEG modifier) using the strain-promoted azide–alkyne cycloaddition reaction^41^. Consistent with DNA modification, the hydrodynamic size of the NPs increased from 48 ± 12 to 62 ± 17 nm post-DNA functionalization (Fig. 2e). Dye-labeled DNA and ultraviolet–visible spectroscopy were used to determine DNA surface coverage (650 strands/particle or ~25 pmol/cm^2^); the ligand density is comparable to gold-based PAEs (~20–40 pmol/cm^2^) and high enough to support cooperativity^15^. Indeed, these MOF PAEs form multivalent, sequence-specific interactions with AuNPs bearing complementary oligonucleotides and generate aggregates that exhibit a characteristic sharp melting transition at 43 °C (Fig. 2f). Finally, the internal pores of the MOF NPs were still accessible post-DNA modification, despite a decrease in BET surface area of 16% and 36% for UiO-66 and PCN-222 (vide infra), respectively (see Supplementary Figs. 11 and 12). The more pronounced reduction in porosity for PCN-222 is attributed to the potential adsorption of DNA molecules in its meso-sized pores (~3.7 nm in diameter) during the DNA functionalization step.

### Colloidal crystal engineering with MOF PAEs

**Fig. 3:**
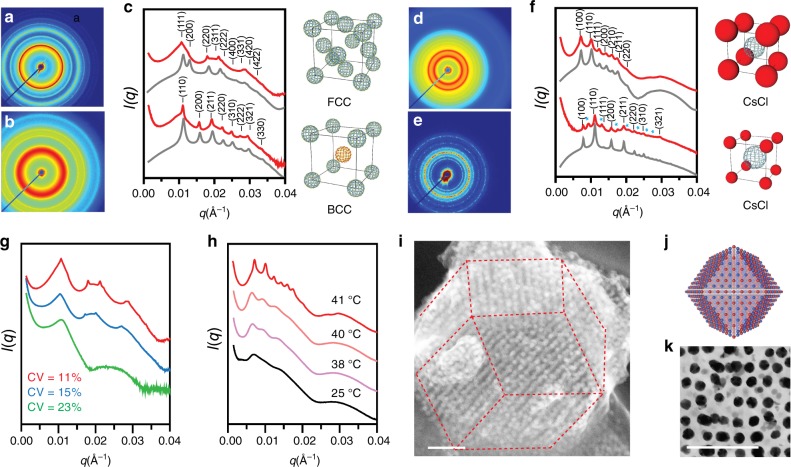
Colloidal crystal engineering with MOF PAEs. **a**–**c** 2D SAXS and radially averaged 1D SAXS patterns (in logarithmic scale) for superlattices formed from **a** 37 nm UiO-66 NPs in an fcc arrangement assembled with self-complementary DNA and **b** binary 37 nm UiO-66 NPs in a bcc arrangement assembled with complementary DNA. Experimental data are shown in red, and simulated scattering patterns are shown in gray. **d**–**f** 2D SAXS and radially averaged 1D SAXS patterns (in logarithmic scale) for CsCl lattices formed from complementary DNA functionalized **d** 37 nm UiO-66 NPs and 20 nm AuNPs (1:1 ratio) and **e** 37 nm UiO-66 NPs and 40 nm AuNPs (1:1 ratio). AB_2_ impurity phase is indicated by blue asterisks. **g** SAXS data of UiO-66 NPs of different uniformity prepared by sucrose-gradient ultracentrifugation: red, 34 ± 4 nm (CV = 11%); blue, 39 ± 6 nm (CV = 15%); green, 47 ± 11 nm (CV = 23%). **h** In situ SAXS monitoring the thermally induced transition of MOF–Au PAE aggregates from the amorphous to crystalline state. **i** Secondary electron STEM image of a single crystalline UiO-66 NP and 40 nm AuNP hybrid superlattices with **j** rhombic dodecahedra crystal habits, and transmission electron image of **k** an 80-nm-thick sample cross-section prepared by ultramicrotomy. All scale bars are 200 nm.

Once the PAEs were characterized, they were studied in the context of colloidal crystal engineering with DNA. We first synthesized face-centered cubic (fcc, Fm$\bar 3$m) and body-centered cubic (bcc, Im$\bar 3$m) colloidal crystals using 37 nm spherical UiO-66 NPs (Fig. 3a–c). Consistent with the complementary contact model^8^, fcc structures form when the MOF NPs present self-complementary DNA sticky ends (5’-GCGC), and bcc structures form when two sets of MOF NPs (of the same size) have complementary DNA sticky ends (5’-AAGGAA, 5’-TTCCTT, see Supplementary Table 2). In a typical experiment, a sample was initially assembled into a disordered state at room temperature and then transformed into a crystalline lattice by thermally melting it (47 °C for 10 min) followed by slow cooling to room temperature at a rate of 0.1 °C/10 min. SAXS experiments verify lattice assignments, and the diffraction peaks suggest the formation of superlattices with micrometer-sized crystalline domains.

Since colloidal crystallization processes are dependent upon NP size dispersity, the crystallinity of the resulting structures should positively correlate with MOF PAE uniformity. To test this hypothesis, three MOF NP samples with increasingly broad size distributions were isolated by the gradient centrifugation method (coefficient of variation (CV) = 11%, 15%, and 23%, respectively), chemically converted into PAEs, and subsequently assembled into fcc-type crystals (Fig. 3g). As shown by SAXS, emerging scattering peaks suggest a clear transition from a relatively disordered state (green trace) to a well-formed fcc lattice (red trace) as MOF PAEs with greater monodispersity were assembled. An amorphous material was obtained for the CV = 23% PAEs (green trace), since the polydisperse building blocks result in more defects, grain boundaries, and lattice strain, all of which inhibit crystallization.

We next explored the formation of binary superlattices composed of MOF and gold PAEs. Based on previously established design rules^8^, complementary DNA functionalized PAEs composed of different core NPs were expected to assemble into CsCl-type (Pm$\bar 3$m) lattices. Indeed, in the case of 37 nm MOF PAEs combined in a 1:1 ratio with complementary 40 nm Au PAEs, CsCl-type lattices emerge (lattice constant = 88.2 nm, Fig. 3d). Owing to a significantly lower scattering cross-section of MOFs, as compared to that of the AuNPs, the relative peak intensities of the simulated SAXS pattern was adjusted by setting the MOF density at 2.1 to match the experimentally derived one-dimensional (1D) SAXS pattern^16^. Next, 37 nm MOF PAEs were combined with complementary 20 nm Au PAEs in a 1:1 stoichiometric ratio, and the resulting CsCl-type lattices (lattice constant = 79.6 nm, Fig. 3e) formed with a small amount of simple hexagonal AlB_2_-type impurities, a similar free energy phase at this size ratio^8^ (sh, P6/mmm: lattice constant, *a* = *b* = 76.5 nm, *c* = 47.6 nm, denoted by blue asterisks in Fig. 3f). Importantly, the DNA-mediated crystallization process can be monitored in situ via SAXS. Indeed, when disordered MOF–Au aggregates are placed in the path of the X-ray beam and gradually heated to 41 °C (slightly below their melting temperature), one could follow by SAXS the formation of a crystalline lattice (Fig. 3h).

The structure and morphology of the crystals were next characterized by scanning transmission electron microscopy (STEM). To obtain samples suitable for electron microscopic imaging, the solution-grown binary MOF–Au superlattices (37 nm UiO-66 NPs assembled with 40 nm AuNPs) were first embedded in silica and then dried under vacuum, a technique that has proven extremely useful for crystals formed from metal and semiconductor NP systems^42^. Remarkably, the MOF–Au superlattices form single crystals with rhombic dodecahedra crystal habits (Fig. 3i, 1–2 μm in edge length). The rhombic dodecahedron is the Wulff polyhedron for a bcc lattice, thereby confirming that the crystallization process is under thermodynamic control^43^. High-magnification cross-section images of a single crystal reveal ordered domains with stacks of individual NPs clearly discernible (Fig. 3k, dark: AuNP, light: UiO-66 NP).

### Building block shape as a structure-influencing factor for MOF NP superlattices

**Fig. 4:**
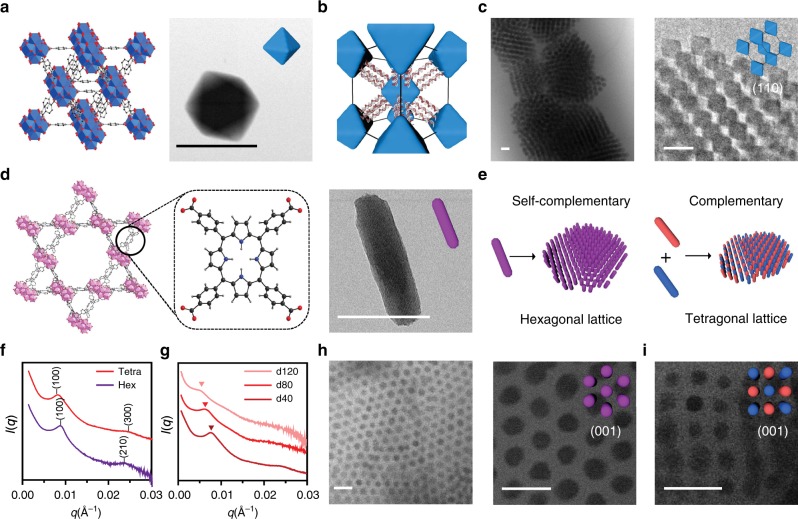
Shape-dependent MOF superlattice assemblies. **a** Crystal structure and bright field (BF) STEM image of an octahedral UiO-66 NP formed by Zr_6_ SBUs and terephthalic acid ligands. **b** A model of a bcc lattice formed with face-to-face oriented UiO-66 PAEs, as such arrangement maximizes DNA hybridization interactions. **c** Cryo-STEM BF images show that octahedral-shaped UiO-66 PAEs with self-complementary DNA linkers assemble into bcc lattices. **d** Crystal structure and BF STEM image of a single PCN-222 nanorod formed by the connection of Zr_6_ SBUs and tetrakis(4-carboxyphenyl)porphyrin linkers (box). **e** Schematic representation of PCN-222 PAEs assembled into two different lattices depending on DNA link design: nanorods functionalized with self-complementary DNA linkers (purple) form a 2D hexagonal superlattice (left), and complementary DNA linkers (red and blue rods) assemble into a 2D tetragonal superlattice (right). **f** 1D SAXS patterns of PCN-222 superlattices with in-plane hexagonal (purple) and tetragonal (red) symmetries. **g** 1D SAXS patterns of PCN-222 tetragonal superlattices with tunable interparticle spacings by changing DNA linker lengths [d40: 77 nm (crimson), d80: 92 nm (red), d120: 109 nm (pink)]. **h** Cryo-STEM BF images show that PCN-222 PAEs with self-complementary DNA linkers close-pack into 2D hexagonal lattices. **i** Cryo-STEM BF image shows that PCN-222 PAEs interconnected by complementary DNA linkers form a 2D tetragonal lattice. All scale bars are 100 nm.

Having shown that DNA and the design rules afforded by the complementary contact model can be used to engineer MOF PAE colloidal crystals, we next explored whether MOF NP shape can be used to access more exotic crystalline states. So far, >70,000 MOFs have been synthesized, exhibiting an extremely rich design space in terms of crystal symmetries and potential crystal habits^44^. Octahedra and nanorods were studied as two representative structures. By combining Zr^4+^ ions with bidentate terephthalic acid or tetravalent porphyrinic acid ligands, UiO-66 octahedra NPs (edge length = 86 ± 10 nm, Fig. 4a) and PCN-222 nanorods (Zr_6_O_8_(H_2_O)_8_(TCPP-H_2_)_2_, TCPP = tetrakis(4-carboxyphenyl)porphyrin, 38 ± 8 nm × 159 ± 25 nm, aspect ratio = 4.3, Fig. 4d) were synthesized, respectively^45^. Upon functionalization with DNA, they were assembled with DNA linkers, annealed, and slowly cooled to room temperature.

Cryogenic STEM bright field (BF) images show that the UiO-66 octahedra with self-complementary GCGC sticky ends crystallize into bcc lattices (Fig. 4c, lattice constant = 180 nm) as opposed to the fcc lattices that form from spheres^46^. This occurs because in the bcc configuration the system can maximize DNA-bonding interactions between neighboring octahedral particles where the facets are aligned in a parallel planar fashion (Fig. 4b)^46^. This effect is due to the anisotropic shape of the PAEs and is expected to become less important as the oligonucleotide length increases and the PAEs begin to behave more like spherical entities^47^. We tested this conclusion by investigating the effect of the DNA linker length on crystallization (by changing duplex DNA linker length, Fig. 1 and Supplementary Table 2). When short DNA linkers were used (one block segment, <40 nm interparticle spacing), structures with small bcc crystalline domains were observed owing to large lattice strain (see Supplementary Fig. 17). When the particles were assembled with intermediate-length DNA linkers (two block segments, ~60 nm interparticle spacing), high-quality bcc crystalline structures were observed (Fig. 4c). The use of long DNA linkers (three block segments, >80 nm interparticle spacing) reduces particle shape anisotropy as well as lattice strain and produces fcc lattices with randomly oriented octahedra. Such observation is in agreement with previous studies on DNA-mediated assembly of gold octahedral particles and reinforces the notion of zones of crystallization and anisotropy related to the relative dimensions of the particle building blocks and DNA-bonding elements^7^.

In contrast, PCN-222 nanorods crystallize into two-dimensional (2D) superlattices with in-plane hexagonal or tetragonal symmetries, depending on the type of DNA linker strands used (Fig. 4d, e). The formation of PCN-222 2D superlattices is confirmed by both SAXS and cryo-STEM experiments (Fig. 4f–i). In the first case, 2D close-packed hexagonal lattices were obtained when self-complementary DNA linkers (GCGC sticky ends) were used, again a consequence of the anisotropic PAE shape (Fig. 4h). In the second case, tetragonal lattices formed when two batches of PCN-222 nanorods, each modified with complementary DNA (5’-AAGGAA, 5’-TTCCTT), were combined and annealed as described above (Fig. 4i). The tetragonal lattice maximizes DNA hybridization between the complementary rod PAE pairs while reducing repulsion between the PAEs with identical sticky ends^47,48^. In addition to demonstrating the utility and versatility of MOF NPs in colloidal crystal engineering with DNA, these 2D architectures are potentially useful as highly tunable separation membranes, since all of the 1D MOF channels are aligned in a parallel configuration, and the lattice parameters of the hexagonal nanorod assemblies can be systematically modulated by varying the length of the DNA linker strands, ranging from 80 to 110 nm (Fig. 4g).

### Photocatalytic activity of the 2D PCN-222 nanorod superlattices

**Fig. 5:**
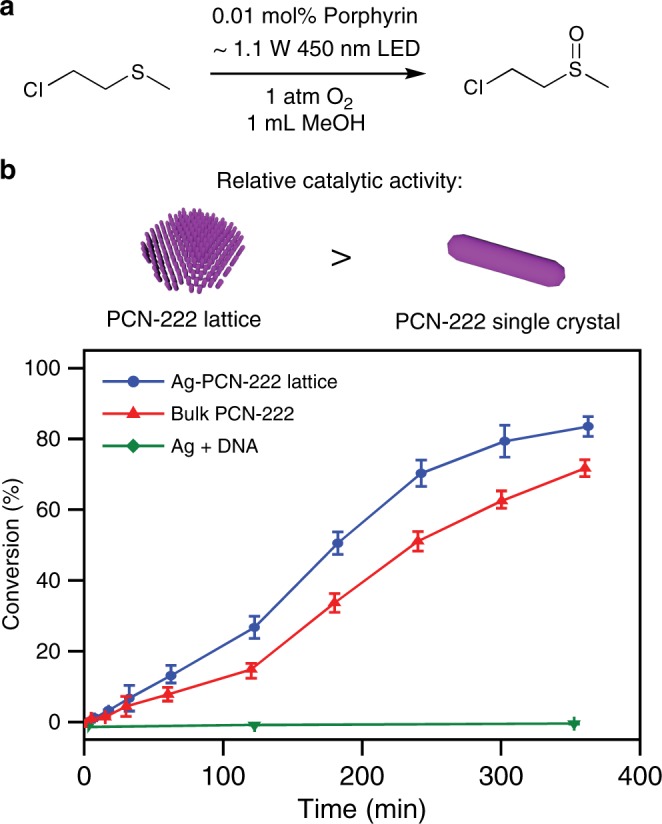
Photocatalytic activity of the 2D PCN-222 nanorod superlattices. **a** Reaction pathway for the oxidation of the mustard gas simulant CEES, using oxygen and a porphyrin photosensitizer. **b** Reaction profiles for the oxidation of CEES using a Ag^+^-stabilized PCN-222 superlattice (blue) and a PCN-222 single crystal (red), respectively (same porphyrin loading). All samples were irradiated with blue light-emitting diodes (LEDs, wavelength_max_ = 450 nm) with equivalent power density. No appreciable catalytic activity was observed for the Ag^+^ ion and single-strand DNA control group (green). The error bars display the standard deviation of measurements for three separately prepared samples.

From a utility standpoint, it is important to determine whether MOF NP functionality is preserved post-DNA functionalization and incorporation in a superlattice. Therefore, the photocatalytic activity of the PCN-222 2D superlattice was investigated and compared with micron-sized PCN-222 single crystals. As a model reaction, the selective partial oxidation of 2-chloroethyl ethyl sulfide (CEES, a chemical warfare agent simulant of mustard gas) was studied because of its implications in detoxification^49–52^. The porphyrin moiety of the MOF ligand was expected to serve as a high quantum yield photosensitizer for the generation of singlet oxygen^53^, which can subsequently oxidize CEES to nontoxic 2-chloroethyl ethyl sulfoxide (CEESO). In a typical reaction, the samples (TCPP ligand loading of 0.01 mol%) were suspended in methanol and purged with O_2_ before the addition of the internal standard and CEES, the reaction progress was monitored by gas chromatography, and the product selectivity was verified by ^1^H nuclear magnetic resonance spectroscopy (see Supplementary Methods). To preserve superlattice structure and MOF porosity, the double-stranded DNA bonds of the PCN-222 superlattices were post-synthetically stabilized with Ag^+^ ion, a method developed to preserve colloidal crystal crystallinity under conditions where DNA bonds are typically denatured^54^. Importantly, the PCN-222 NP superlattice shows selective conversion of CEES to CEESO (see Supplementary Fig. 26) and a higher catalytic conversion efficiency as compared to bulk PCN-222 crystals (Fig. 5). This observation could be attributed to faster substrate diffusion kinetics induced by the smaller MOF particle sizes and a higher photosensitization efficiency associated with the 2D superlattice, which supports a larger light absorption cross-section than 3D crystals and thus results in a greater penetration depth^52,55,56^.

## Discussion

In summary, we have reported a general method for the preparation of PAEs from MOF NPs and DNA, providing access to a diverse set of colloidal crystals spanning numerous crystal symmetries with tunable lattice parameters. When combined with all that is known about MOF NP shape control, these experiments suggest that in addition to MOF NPs being a new compositionally diverse set of building blocks, with which colloidal crystal properties can be tuned, particle shape-induced bonding interactions with MOF PAEs will be extremely useful for engineering crystal outcomes^47^. More importantly, the ability to precisely design 2D arrays and 3D MOF superlattices should allow for the development of designer materials where sensing, catalytic, and light-harvesting modalities can be systematically interfaced within crystalline structures with sub-nm precision.

## Methods

Descriptions of the methods for MOF NP syntheses, DNA functionalization, superlattice crystallization, electron microscopy, and SAXS characterization of assemblies are available in Supplementary Information.

## Supplementary information

Supplementary Information

## Data Availability

The datasets generated during and/or analyzed during the current study are available from the corresponding author upon reasonable request.
