# Supplementary material for: Colloidal crystal engineering with metal–organic framework nanoparticles and DNA
Source: Nat Commun. 2020 May 19;11:2495. doi: 10.1038/s41467-020-16339-w (PMC7237412; doi:10.1038/s41467-020-16339-w)
Supplement: Supplementary file 1 — Supplementary Information [file 41467_2020_16339_MOESM1_ESM.pdf]

Supplementary Information

for

Colloidal Crystal Engineering with Metal-Organic Framework Nanoparticles and DNA

Wang et al.

Supplementary Methods

Materials

All reagents, unless otherwise stated, were obtained from commercial sources and were used without further purification. All oligonucleotides used in this work were synthesized on a solid-support MM12 synthesizer with reagents purchased from Glen Research. The water used in all experiments was ultrapure deionized (DI) grade (18.2 MΩ·cm resistivity), obtained from a Milli-Q Biocel system (Millipore).

Synthesis of heterobifunctional PEG ligands

Synthesis of diethyl 2-azidoethylphosphonate

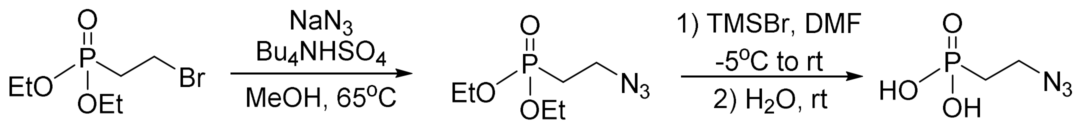

Diethyl 2-bromoethylphosphonate (250 mg, 1.0 mmol) was reacted with sodium azide (260 mg, 4.0 mmol) and tetrabutylammonium hydrogensulfate (510 mg, 1.5 mmol) in 4 mL methanol (MeOH) at 65 ºC for 18 h. After cooling to room temperature, the solvent was evaporated and diluted with diethyl ether, and filtered through Celite. The filtrate was washed with water, and the organic layer was dried over sodium sulfate to yield diethyl 2-azidoethylphosphonate (90%).

Synthesis of 2-azidoethylphosphonic acid

In 0.8 mL of *N,N*-dimethylformamide (DMF), diethyl 2-azidoethylphosphonate (120 mg, 0.57 mmol) was reacted with 4 equivalents of bromotrimethylsilane (350 mg, 2.3 mmol) at -5 ºC. After warming to room temperature, the product was filtered and washed with water and dried to yield 2-azidoethylphosphonic acid (80%).

Synthesis of Phosphate-PEG$_4$-N$_3$

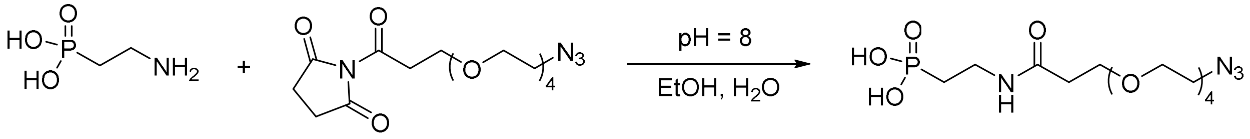

The pH of a 5 mL aqueous solution of 2-aminoethylphosphonic acid (12.5 mg, 0.10 mmol) was adjusted to 8 with 1 M sodium hydroxide, and 15-Azido-4,7,10,13-tetraoxapentadecanoic acid succinimidyl ester (azido-PEG$_4$-NHS ester from Click Chemistry Tools, 77.6 mg, 0.2 mmol) dissolved in 5 mL of DMF was added. The reaction mixture was stirred at 4 ºC for 4 h and incubated overnight at room temperature. After the reaction, the solvent was removed by lyophilization, and the product was used as a mixture to functionalize MOF nanoparticles (NPs) without further purification. In our study, approximately 80% 2-aminoethylphosphonic acid was converted to phosphate-PEG$_4$-N$_3$.

Synthesis of Phosphate-PEG$_{110}$-N$_3$

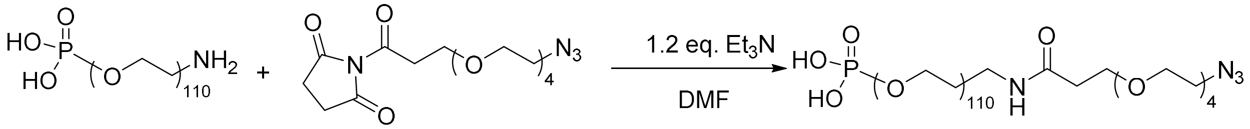

In a 25 mL glass vial, phosphate-PEG$_{5k}$-NH$_2$ (200 mg, 40 μmol) and azido-PEG$_4$-NHS ester (120 mg, 320 μmol) were dissolved in 10 mL dry DMF, and triethyl amine (12 mg, 120 μmol) was added to the solution. The mixture was stirred at room temperature overnight. DMF was removed by lyophilization to yield a yellow gel. The yellow gel was dissolved in water and passed through a 3 kDa cut-off centrifuge filter three times (3,000 rpm, 20 min) to remove excess azido-PEG$_4$-NHS. The product was lyophilized to yield a light yellow solid (~50%).

Synthesis and characterization of MOF NPs

Synthesis of spherical UiO-66 [Zr$_6$O$_4$(OH)$_4$(BDC)$_{12}$] NPs

Spherical UiO-66 NPs (37 ± 8 nm) were synthesized via an acetic acid modulated solvothermal reaction.[1] 1,4-benzenedicarboxylic acid (25 mg, 0.25 mmol) was dissolved in 0.5 mL of DMF. In a separate vial, zirconyl chloride octahydrate (21 mg, 66 μmol) was dissolved in 3 mL of DMF. The two solutions were mixed together in a 10 mL scintillation vial, and 0.5 mL acetic acid was added to the reaction mixture. After brief sonication, the solution was heated at 90 ˚C in an oven

for 6 h to yield uniform UiO-66 NPs. The NPs were collected by centrifugation (12000 rpm, 30 min), followed by solvent exchange with DMF, and stored in anhydrous DMF.

Synthesis of octahedral UiO-66 [$Zr_6O_4(OH)_4(BDC)_{12}$] NPs

Octahedral UiO-66 NPs (86 ± 10 nm) were synthesized via an acetic acid modulated solvothermal reaction.[1] 1,4-benzenedicarboxylic acid (10 mg, 60 µmol) was dissolved in 0.2 mL of DMF. In a separate vial, zirconyl chloride octahydrate (21 mg, 66 µmol) was dissolved in 3 mL of DMF. The two solutions were combined in a 10 mL scintillation vial, and 0.5 mL acetic acid was added to the reaction mixture. After brief sonication, the solution was heated at 90 ˚C in an oven for 5 h to yield uniform UiO-66 NPs. The NPs were collected by centrifugation (10000 rpm, 20 min), followed by solvent exchange with DMF, and stored in anhydrous DMF.

Synthesis of PCN-222/MOF-545 [$Zr_6O_8(H_2O)_8(TCPP\text{-}H_2)_2$] NPs

The synthesis of PCN-222/MOF-545 nanorods (38 ± 8 × 159 ± 25 nm, aspect ratio = 4.3) was based on a literature reported method with minor modifications.[2] Zirconyl chloride octahydrate (38 mg, 0.12 mmol) and tetrakis(4-carboxyphenyl)-porphyrin (6.5 mg, 0.0082 mmol) were dissolved in DMF (16 mL) in a 22 mL borosilicate vial with a Teflon-lined cap. Dichloroacetic acid (0.30 mL, 3.0 mmol) was added, and the resulting solution was heated at 130 °C in an oven for 18 h to afford dark purple rod-shaped NPs and a yellow mother liquor. The NPs were collected by centrifugation (12000 rpm, 30 min), followed by solvent exchange with DMF, and stored in anhydrous DMF.

Powder X-ray diffraction (PXRD)

The crystallinity of the synthesized MOF NPs and MOF programmable atom equivalents (PAEs) were confirmed by PXRD. PXRD patterns were recorded on Rigaku Smartlab instrument using Nickel-filtered Cu-Kα radiation (λ = 1.5418 Å) with an accelerating voltage and current of 45 kV and 160 mA, respectively.

Electron microscopy experiments

Transmission electron microscopy (TEM) and scanning electron microscopy (SEM)

MOF NPs were analyzed using a Hitachi HD-2300 scanning transmission electron microscope (STEM) in either secondary electrons (SE) or transmitted electron (TE) mode with an accelerating voltage of 200 kV. Samples were dispersed onto TEM grids by drop-casting a dilute ethanol solution containing MOF NPs or MOF PAEs directly onto TEM grids. The average particle size for each synthesis was determined by measuring the edge length of more than 100 particles from multiple syntheses under analogous synthetic conditions.

Cryo-STEM imaging

4 μL of samples were pipetted onto glow discharged lacey carbon 200 mesh Cu grids (EMS Cat. # LC200-CU-100), blotted for 5 s, and plunge-frozen in liquid ethane with an FEI Vitrobot Mark III. The samples were then loaded into a Gatan 626.6 Cryo Transfer Holder kept at -165 °C and imaged in a Hitachi HD2300 cFEG STEM at 200 kV utilizing TE phase contrast and high angular annular dark field (HAADF) Z–contrast.

Sucrose-gradient ultracentrifugation

To improve the uniformity of MOF NPs, an aqueous solution of UiO-66 NPs was concentrated and centrifuged at 20,000 rcf on a continuous sucrose gradient. The range of gradient density and duration of centrifugation step varies depending on the size and density of the MOF NPs. For instance, to separate 300 μL of 37 nm UiO-66 NPs, 10 mL of a 10%-50% gradient mixture was used, and 40 min centrifugation resulted in satisfying separation (Table S1). The layer of NPs was recovered from the gradient with a syringe and washed twice through a 50 kDa Amicon centrifugal filter tube in order to clean the sample from sucrose and to dissolve it in desired medium to prepare the samples for characterization and polymer/DNA conjugation.

Synthesis of oligonucleotides

Oligonucleotides were synthesized using a Mermaid MM12 DNA synthesizer (Bio Automation) on a standard controlled pore glass (CPG) solid phase support. All oligonucleotides were deprotected under conditions recommended by the manufacturer and purified by reverse phase high performance liquid chromatography (HPLC). Characterization and oligonucleotide concentration were determined by MALDI-TOF mass spectrometry and UV-vis spectroscopy, respectively. A complete list of oligonucleotides synthesized can be found in Table S2.

DNA functionalization of NPs and assembly of colloidal crystals

DNA functionalization of MOF NPs

Phosphate-PEG-N_3 linker functionalization

In a 1.5 mL Eppendorf tube, 10 mg phosphate-PEG_{5k}-N_3 linker (~1×10^{18} polymer) was dissolved in 100 µL DMF (~ 20 mM). In a separate tube, 33 pmol of 37 nm UiO-66 NPs (~2×10^{13} particles) was dispersed in 1 mL DMF and 100 µL linker solution (10 mg linker) was added. Similarly, 14.1 pmol of PCN-222 nanorods (~8.5×10^{12} particles) were dispersed in 1 mL DMF and 50 µL linker solution (5 mg linker) was added. The mixture was sonicated and incubated on a thermal shaker at 750 rpm and 25 ºC for 48 h. The functionalized NPs were first washed with DMF to remove excess PEG_{5k} ligands (centrifugation: 15,000 rpm, 45 min), and then sequentially washed with a 1:1 DMF/water mixtures, and twice with water. The NP samples were concentrated to ~ 50 nM and stored in DI water.

DNA functionalization

In a typical DNA functionalization experiment, azide modified MOF NPs were dispersed in water and functionalized with DBCO-TEG modified DNA (MOF-Bound DNA). To functionalize UiO-66 NPs, 300 nmol of DNA was dissolved in 1 mL water (0.3 mM, 1.8×10^{17} DNA), to which 33 pmol of 37 nm UiO-66 NPs (33 nM, ~ 2×10^{13} particles) were added. Similarly, to functionalize 160 nm PCN-222 nanorods, 200 nmol of DNA was dissolved in 1 mL water (0.2 mM, 1.2×10^{17} DNA), to which 2 pmol of PCN-222 nanorods (2 nM, ~ 1.5×10^{12} particles) were added. The DNA and MOF NPs were incubated on a thermal shaker overnight at 25 ºC at 750 rpm. Sodium chloride (NaCl) was slowly added to the solution to a final concentration of 0.5 M (over 8 h), which reduces

electrostatic repulsion between negatively charged neighboring oligonucleotide strands, allowing one to achieve high surface densities of DNA. The total incubation time should be > 48 h, and gentle sonication should be applied occasionally to prevent aggregation of particles. Excess oligonucleotides were removed by centrifugation (5 × 5,000 rpm, 10 min), followed by resuspension of the MOF PAEs in water.

DNA functionalization of gold nanoparticles (AuNPs)

20 nm and 40 nm citrate-capped spherical AuNPs (Ted Pella) were used as received with no further modification. DNA-functionalization of AuNPs with thiol-modified oligonucleotides was carried out according to literature procedures.[3] Briefly, 100 nmol of the AuNP-bound assembly strand (Table S2, sequence AuNP-bound A) were treated with a solution of 100 mM dithiothreitol (DTT, pH = 8) for approximately 1 h and subsequently purified using Nap-5 size exclusion columns (GE Healthcare) to remove residuals. The surfactant, sodium dodecyl sulfate (SDS), was added to the solution of AuNPs to bring the final surfactant concentration to 0.02 vol%, followed by the addition of purified thiolated DNA (approximately 4-5 nmol DNA per mL of AuNPs). A 5 M solution of NaCl was slowly added to the NP solution over the next several hours in a "salt aging" process to increase the density of DNA on the particle surface by shielding against electrostatic repulsion between strands. After bringing the final salt concentration to 0.5 M NaCl, the particles were allowed to sit overnight, followed by three rounds of purification with centrifugation (4,000 – 15,000 rpm; times varied from 10-60 min depending on the NP size), removal of supernatant, and resuspension of the NP pellet in water to remove any unreacted DNA, salt, and surfactant. After removal of the supernatant following the final round of centrifugation, salt was added to the purified particles to bring the final concentration to 0.4 M NaCl, which is the salt concentration at which all subsequent assembly reactions took place.

Colloidal crystal assembly experiments

Synthesis of MOF fcc superlattice

In a typical experiment, 2000 equivalents of self-complementary linker were added to 50 nM solutions of DNA functionalized 37 nm UiO-66 NPs in 0.02% SDS and 0.5M NaCl. The addition of self-complementary DNA linkers resulted in the formation of aggregates that settled down

within minutes. Samples were heated to a few degrees above their melting temperature (55 ºC) and cooled at a rate of 0.01 ºC/min to 20 ºC using a ProFlexTM PCR system (Applied Biosystems).

Synthesis of MOF-MOF bcc superlattice

In a typical experiment, 1000 equivalents of complementary linker A and 1000 equivalents of complementary linker B were added to 20 nM solutions of DNA functionalized 37 nm UiO-66 NP A and UiO-66 NP B (1:1 ratio) in 0.02% SDS and 0.5 M NaCl, respectively. The mixtures were incubated for 20 min on a thermal shaker to allow linker hybridization, followed by the combination of 50 μL of each solution, which resulted in the formation of aggregates that settled down within minutes. Samples were heated to a few degrees above their melting temperature and cooled at a rate of 0.01 ºC/min to 20 ºC using a PCR system.

Synthesis of MOF-Au CsCl hybrid superlattice

In a typical experiment, 2000 equivalents of complementary linker A and 1000 equivalents of complementary linker B were added to 10 nM solutions of DNA functionalized 40 nm AuNPs and 37 nm UiO-66 NPs (1:1 ratio) in 0.02% SDS and 0.5 M NaCl, respectively. The mixtures were incubated for 20 min on a thermal shaker to allow for linker hybridization, followed by the combination of 50 μL of each solution, which resulted in the formation of aggregates that settled down within minutes. Samples were heated to a few degrees above their melting temperature and cooled at a rate of 0.01 ºC/min to 20 ºC using a PCR system.

N_2 sorption measurements

N_2 adsorption isotherm measurements were performed on a Micromeritics Tristar II 3020 (Micromeritics, Norcross, GA) at 77K. The sample was transferred to the oven-dried sample tube and heated to 150 °C under vacuum. Surface areas were estimated by applying the Brunauer–Emmett–Teller (BET) equation (Fig. S11 and S12).[45]

Quantification of DNA surface coverage

UV-vis spectroscopy

UV-vis spectroscopy was performed on a Cary 5000 UV-vis spectrometer (Agilent) with 1 cm quartz optical cells were used for the measurements. The temperature was regulated with a Peltier heat pump attached to a six-cell holder. The surface DNA coverage of the MOF PAEs was determined by UV-vis spectroscopy with Tamra labeled DNA.

Inductively coupled plasma-optical emission spectroscopy (ICP-OES)

DNA coverage of each MOF was quantified based on their NP surface area and NP molar concentration. With the radius/edge length of each MOF NP obtained from TEM, the surface area of each NP was calculated based on geometric approximations: spheres for 37 nm UiO-66 NPs and rod for PCN-222 nanorods. The molar concentration of each MOF NP sample was obtained by ICP-OES analysis of the Zr contents of these MOF samples in addition to crystallographic information; the number of metal atoms per NP can be calculated for a given size MOF NPs. ICP-OES analysis was carried out on a Thermo iCap 7600 ICP-OES instrument with an automated sample changer. MOF NP samples were dispersed in DMF (1 mL), and 10 µL of the MOF sample was added to HNO_3 (990 µL). The samples were heated at 60 ˚C for 15 h for full digestion.

$^{31}P\{^1H\}$ magic angle spinning solid state nuclear magnetic resonance (MAS NMR) spectroscopy

$^{31}P\{^1H\}$ MAS NMR spectroscopy was performed on a Varian 400 MHz VNMRS system (512 scans, 5 s recycle time, and 10,000 Hz spin rate).

Small angle X-ray scattering (SAXS) studies

Instrumentation and methods

SAXS characterization was carried out at the sector 5 DuPont-Northwestern-Dow Collaborative Access Team (DND-CAT) beamline of Argonne National Laboratory's Advanced Photon Source (APS). X-rays of wavelength 1.24 Å (10 keV) were used, and the system was calibrated using silver behenate as a standard. Two sets of slits were used to define and collimate the X-ray beam; parasitic scattering was removed via a pinhole. Typical exposure times varied between 0.1 and 0.5

s, depending upon the sample. The scattered X-rays were collected with a CCD area detector and 1-dimensional scattering data were obtained from radial averaging of the 2-dimensional data to obtain plots of scattering intensity as a function of the scattering vector q. One-dimensional SAXS data were indexed using Matlab to determine the crystallographic symmetry and the lattice parameter.[6]

Calculation of interparticle distances and lattice parameters

For the assembly parameters presented in the previous section, all relevant interparticle distances and lattice parameters were calculated using the SAXS data. The distance between NP nearest neighbors within a NP superlattice can be determined as: $d_{NP} = (\frac{1}{10})(\frac{C}{q_0})$, where d_{NP} is the distance in nm between two NP nearest neighbors, q_0 is the position of the initial scattering peak in 1/Å, and C is a constant that correlates the distance between two nanoparticle nearest neighbors and the distance between the (*hkl*) planes associated with the first scattering peak. Values of C are summarized in Table S3. To ensure that the lattice parameters and interparticle distances matched those calculated using equation, modeled SAXS patterns were generated with PowderCell and compared to the experimental data; this ensured that all diffraction peaks matched with the calculated lattice parameter values.

Immobilization of MOF-Au hybrid superlattice in solid phase for imaging

Silica encapsulation of MOF-Au binary superlattice

Immobilization of MOF-AuNP hybrid superlattice from colloidal solution to the solid-state was achieved by silica encapsulation.[7] Briefly, *N*-trimethoxysilylpropyl-*N,N,N*-trimethylammonium chloride (TMSPA) (2 µL, 7.2 µmol) and 10 mg polyvinyl pyrrolidone (PVP, M.W. = 58,000, dissolved in 100 µL of 0.5 M NaCl) was added to the superlattices in 0.5 mL of 0.5 M NaCl solution, and the mixture was stirred for approximately 30 min before the addition of triethoxysilane (TES) (4 µL, 21.7 µmol). The suspension was vigorously stirred for 24 h at room temperature, followed by purification to remove excess silica by three rounds of centrifugation and resuspension in water. For STEM analysis, the samples were solvent exchanged with ethanol and drop-casted onto a TEM grid.

Resin embedding of silica embedded superlattice samples

For TEM analysis of the superlattice cross section, silica-encapsulated superlattice samples were resin embedded. The detailed procedure for resin embedding was adopted from a previous literature report.[8] Briefly, ~ 5 mg of silica-encapsulated superlattices was first embedded within 0.2 mL of 4% gelatin. The gelatin sample was dehydrated upon immersion in anhydrous ethanol solutions of increasing concentration (30%→50%→70%→80%→90%→100%). Next, the 100% ethanol was solvent exchanged with acetone twice for 10 min. In acetone, the gelatin was embedded in EMBed-812 resin (Electron Microscopy Sciences) following standard protocols provided by the manufacturer. The samples were placed at 65 °C for 16-20 h to polymerize and solidify the resin. The resin containing superlattice was sectioned into 100 nm slices for imaging.

Preparation of PCN-222 superlattice stabilized with Ag^+

The Ag^+-stabilized PCN-222 superlattice was prepared following literature report.[9] Briefly, the assembled PCN-222 superlattice was first solvent exchanged with aqueous 0.5 M $NaClO_4$ solution (containing 0.01 wt% SDS) for three times to remove any Cl^- ions. Next, 0.5 M $AgNO_3$ aqueous solution was rapidly added to the tube containing the superlattice, and the mixture was incubated for 12 h at room temperature in the dark. Finally, the Ag^+-stabilized crystals were solvent exchanged with water and methanol for three times.

Catalytic photooxidation of CEES with PCN-222 2D superlattice

Quantification of porphyrin content in MOF samples

The porphyrin contents of the PCN-222 nanorod superlattice and bulk PCN-222 were quantified by UV-vis spectroscopy. In a typical experiment, MOF samples were digested in 0.1 M NaOH to dissolve the framework, and the absorbance at 420 nm (Soret band of H_4TCPP) of the supernatant was measured and used to quantify the porphyrin content with a calibration curve.

Thermal and chemical stability studies of Ag-stablized PCN-222 superlattice

The Ag^+-stabilized crystals were solvent exchanged with water and methanol for three times each, prior to catalytic oxidation experiment. The chemical and thermal stability of these modified

superlattices were confirmed by PXRD studies. The Ag-stabilized PCN-222 superlattice is observed to be thermally stable at 150 °C for 48 h (Fig. S21), and chemically stable in a range of conditions, as evidenced by their preserved crystallinity (Fig. S22 and S23), which are comparable to the pristine MOF NPs.

CEES Oxidation

The partial oxidation of 2-chloroethyl ethyl sulfide (CEES) to 2-chloroethyl ethyl sulfoxide (CEESO) was performed under similar conditions reported in literature.[10-11] In a typical experiment, MOF samples were loaded in a microwave vial containing 1 mL of methanol. The vial was sealed and purged with O_2 for 20 min, followed by addition of the internal standard and CEES amounting to a porphyrin loading of 0.01 mol %. Two blue LEDs (λ_{max} = 450 nm, full width half maximum = 18 nm), ~5 cm apart and set to 1.1 W/cm^2, were used to irradiate the sample. CEES conversion data points were collected at regular intervals of irradiation. Conversion of CEES was calculated relative to the internal standard by gas chromatography (GC-FID). The stability of the catalysts was confirmed by comparing PXRD spectra of the catalyst before and after 5 catalytic cycles (Fig. S24). The amount of porphyrin leached from the catalyst was quantified to be less than 2% by weight, according to the porphyrin content present in the supernatant as compared to the isolated solid via UV-vis spectroscopy (Fig. S25).

CEES oxidation product selectivity

The sample product selectivity was verified by ^{1}H NMR spectroscopy. No 2-chloroethyl ethyl sulfone ($CEESO_2$) toxic byproduct was observed.

Supplementary Figures

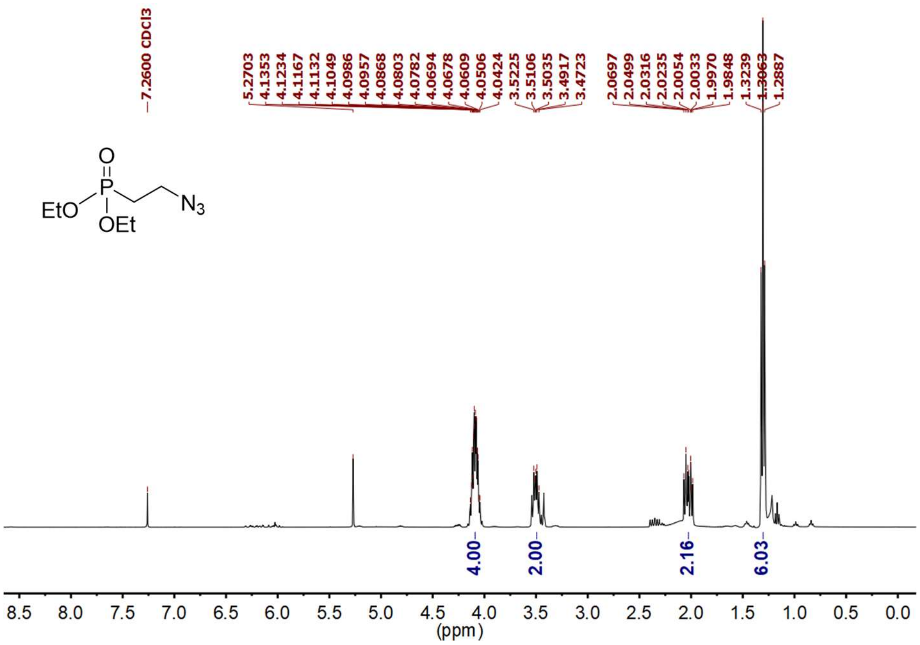

Supplementary Figure 1. ^{1}H NMR spectrum of diethyl 2-azidoethylphosphonate. The trace amount of impurities was carried to the next step without further purification, which disappeared post-hydrolysis.

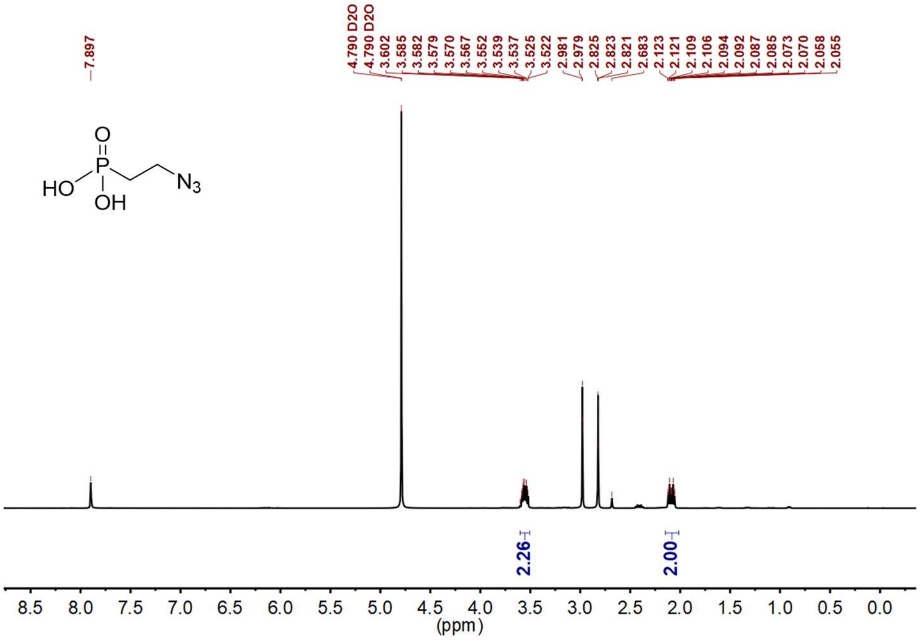

Supplementary Figure 2. ^{1}H NMR spectrum of 2-azidoethylphosphonic acid.

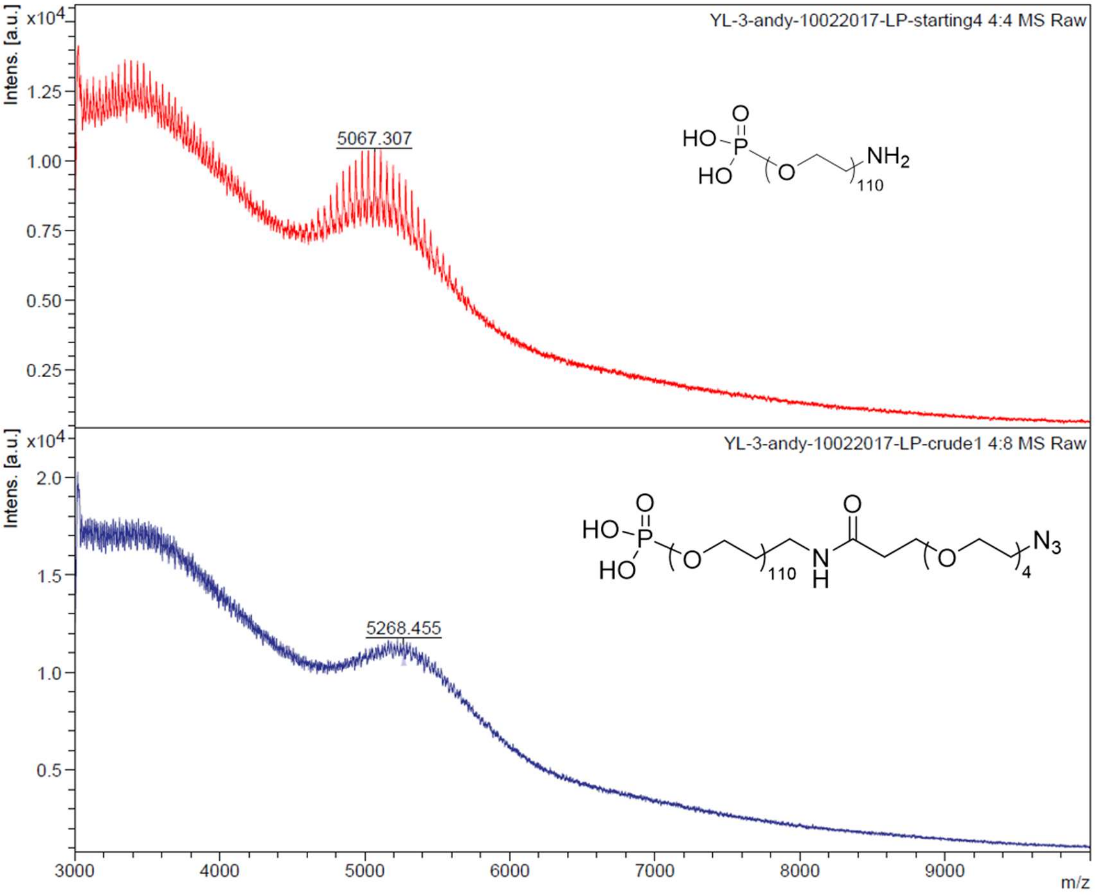

Supplementary Figure 3. Matrix-assisted laser desorption/ionization time-of-flight (MALDI-TOF) spectra of the PEG_{5k} starting material (red curve) and phosphate PEG_{5k} ligand (blue curve).

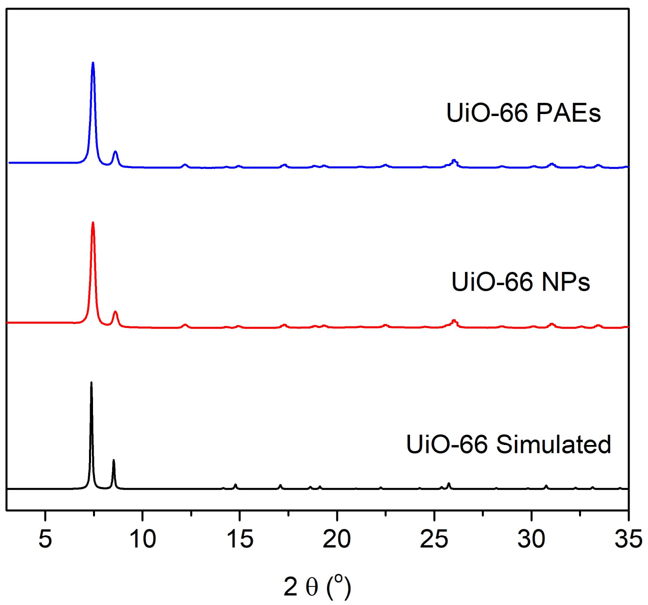

Supplementary Figure 4. Simulated PXRD pattern of UiO-66 (black) and patterns of UiO-66 NPs before (red) and after DNA functionalization (blue).

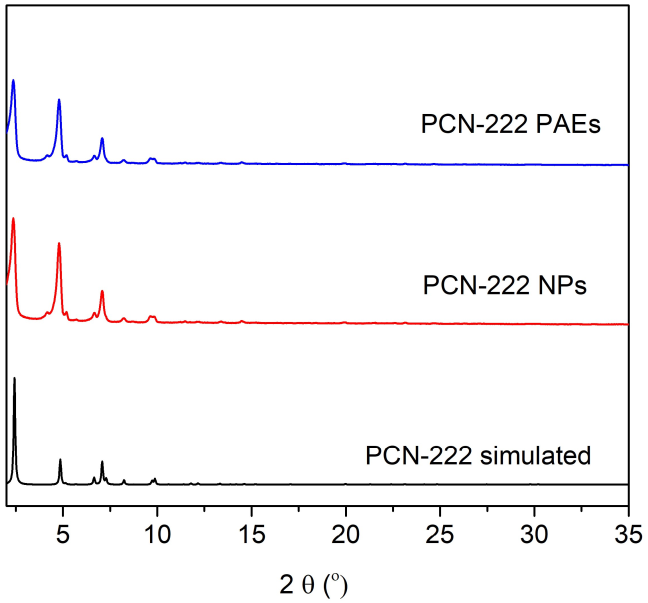

Supplementary Figure 5. Simulated PXRD pattern of PCN-222 (black) and patterns of PCN-222 nanorods before (red) and after DNA functionalization (blue).

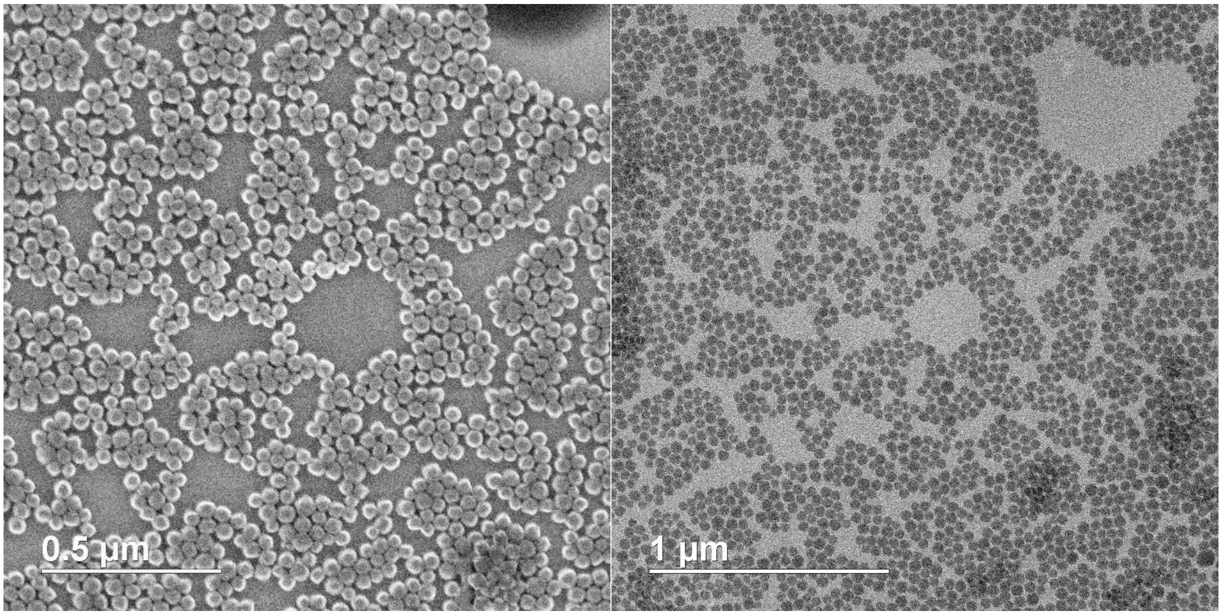

Supplementary Figure 6. SEM (left) and TEM (right) images of spherical UiO-66 NPs (37 ± 4 nm).

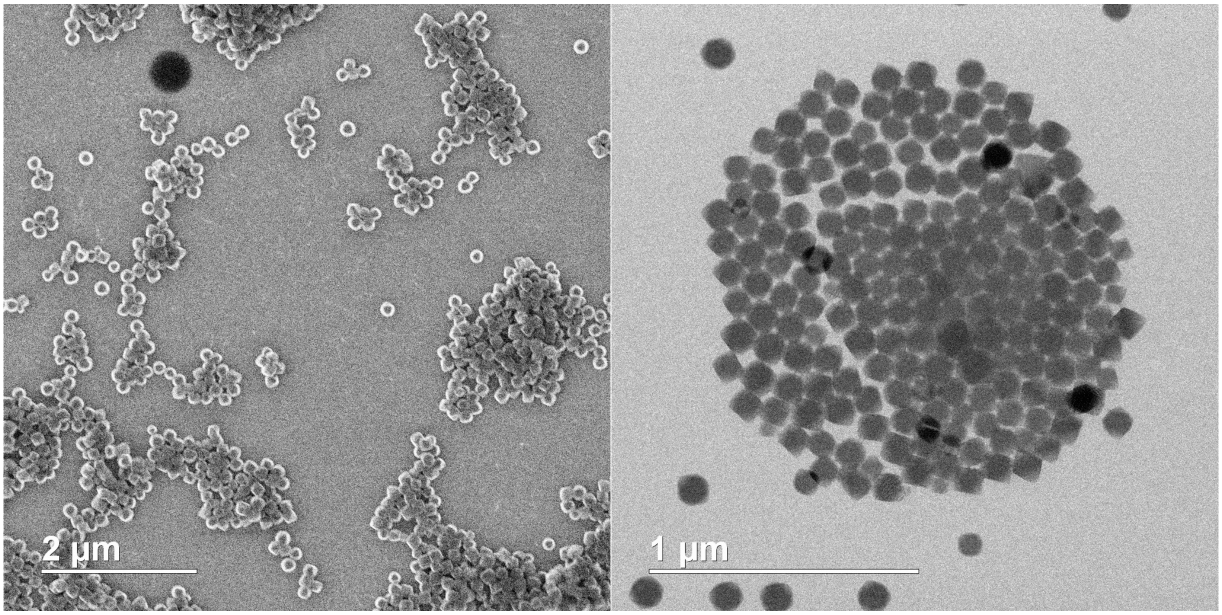

Supplementary Figure 7. SEM (left) and TEM (right) images of octahedral UiO-66 NPs (86 ± 10 nm).

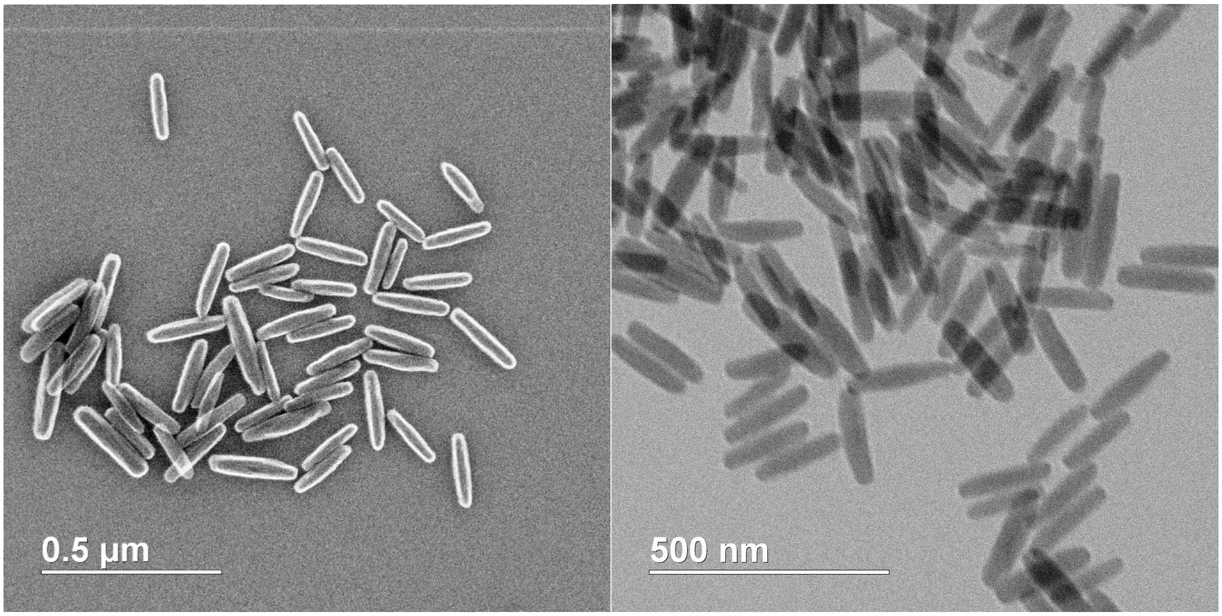

Supplementary Figure 8. SEM (left) and TEM (right) images of PCN-222 nanorods (37 ± 8 × 159 ± 25 nm).

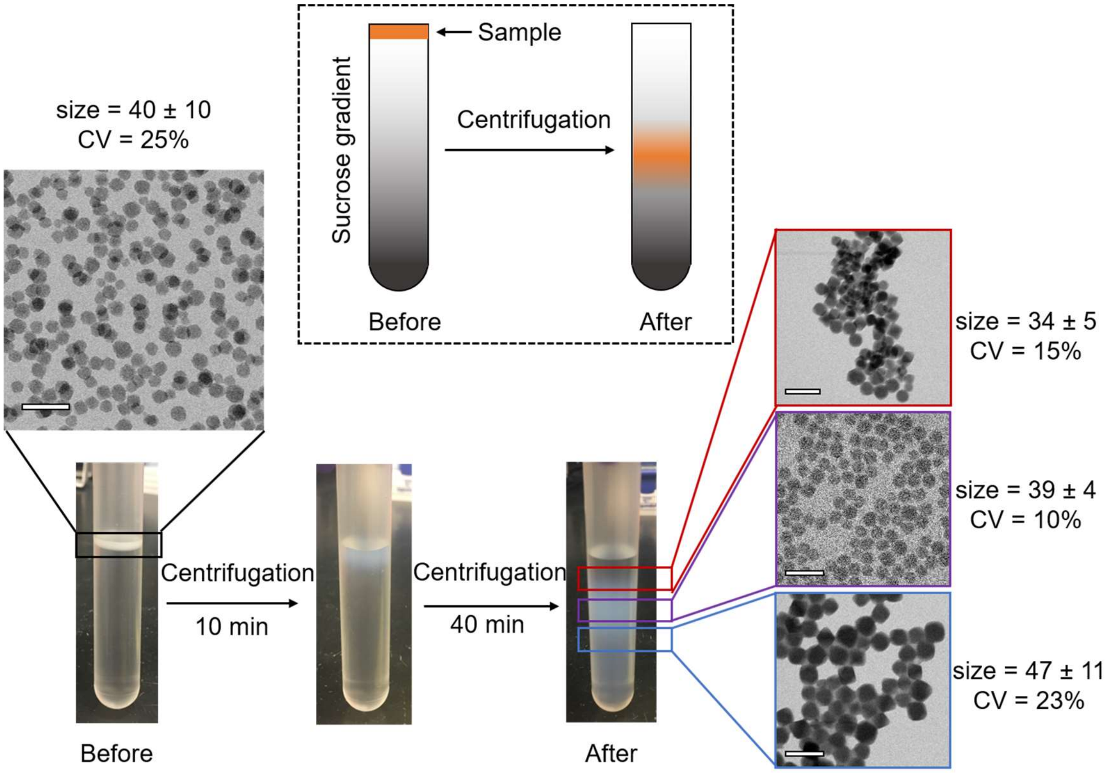

Supplementary Figure 9. Schematic representation of the sucrose gradient based ultra-centrifugation method for UiO-66 NPs. All scale bars are 100 nm.

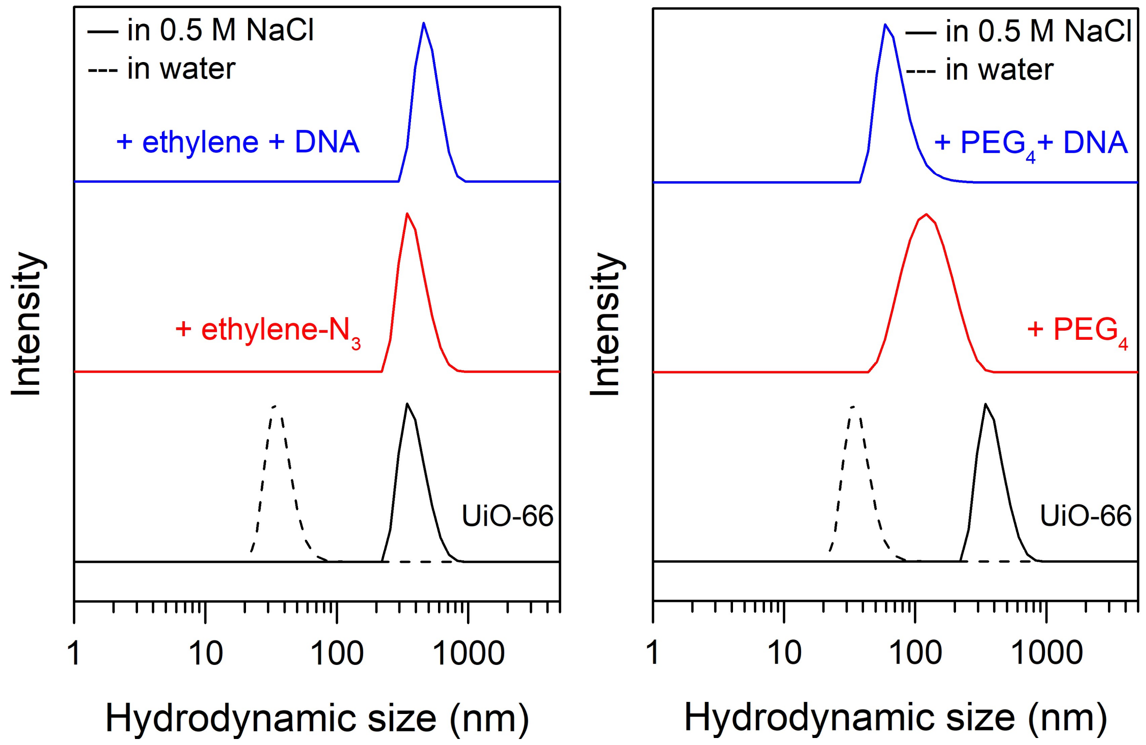

Supplementary Figure 10. Dynamic light scattering intensity analyses of linker (red) and DNA (blue) functionalized UiO-66 NPs in water (dashed) and 0.5 M NaCl (solid). Significant increase in hydrodynamic size and size distribution were observed for both samples, suggesting poor colloidal stability.

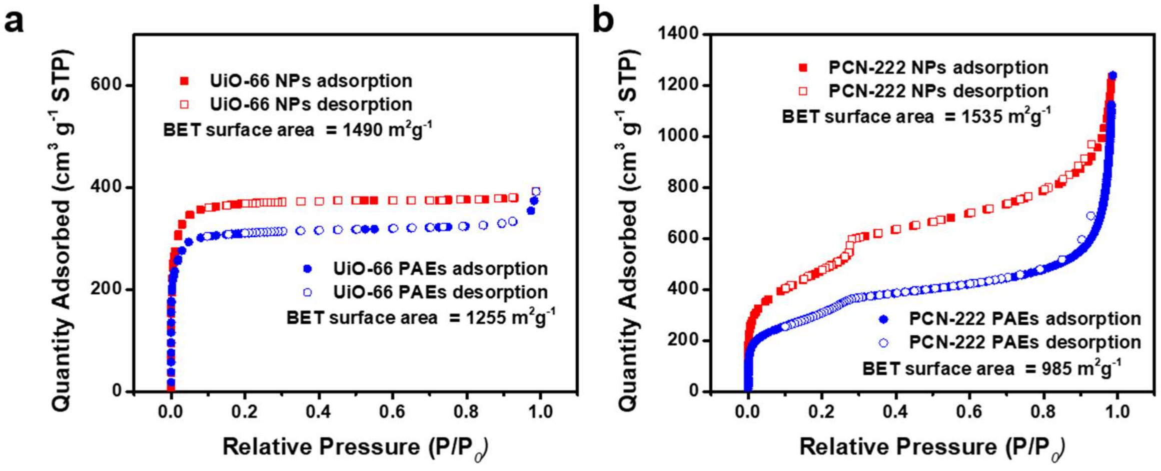

Supplementary Figure 11. N_2 adsorption isotherms of a) UiO-66 NPs (red) and UiO-66 PAEs (blue) and b) PCN-222 NPs (red) and PCN-222 PAEs (blue).

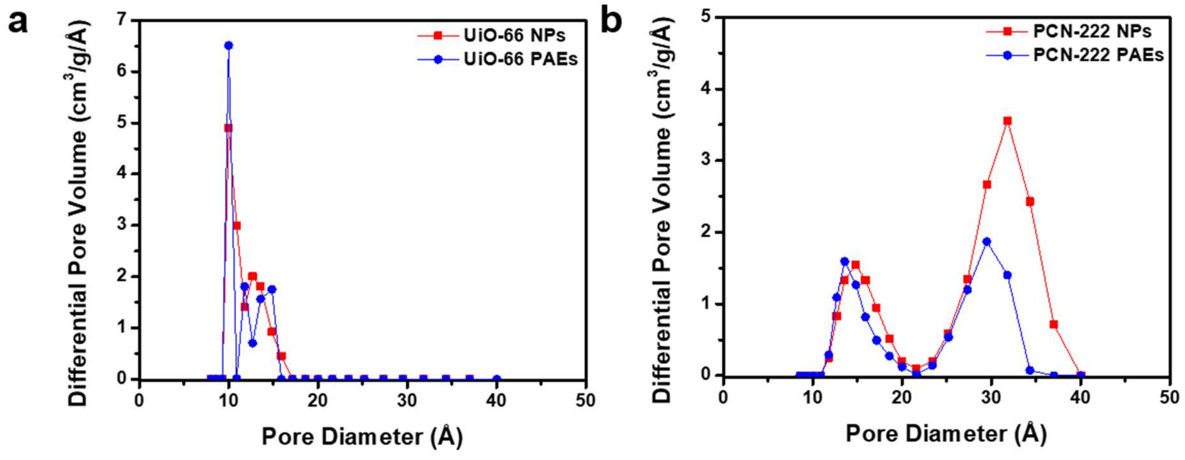

Supplementary Figure 12. Density functional theory pore size distribution analyses of a) UiO-66 NPs (red) and UiO-66 PAEs (blue), b) PCN-222 NPs (red) and PCN-222 PAEs (blue).

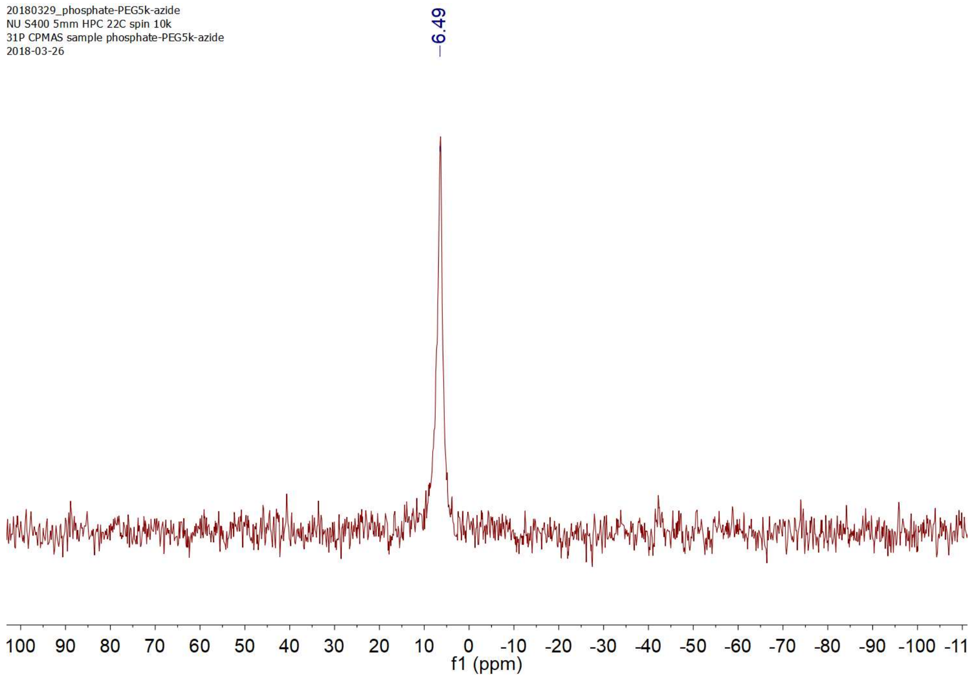

Supplementary Figure 13. $^{31}P\{^{1}H\}$ MAS NMR spectrum of the phosphate-PEG_{5k}-azide linker.

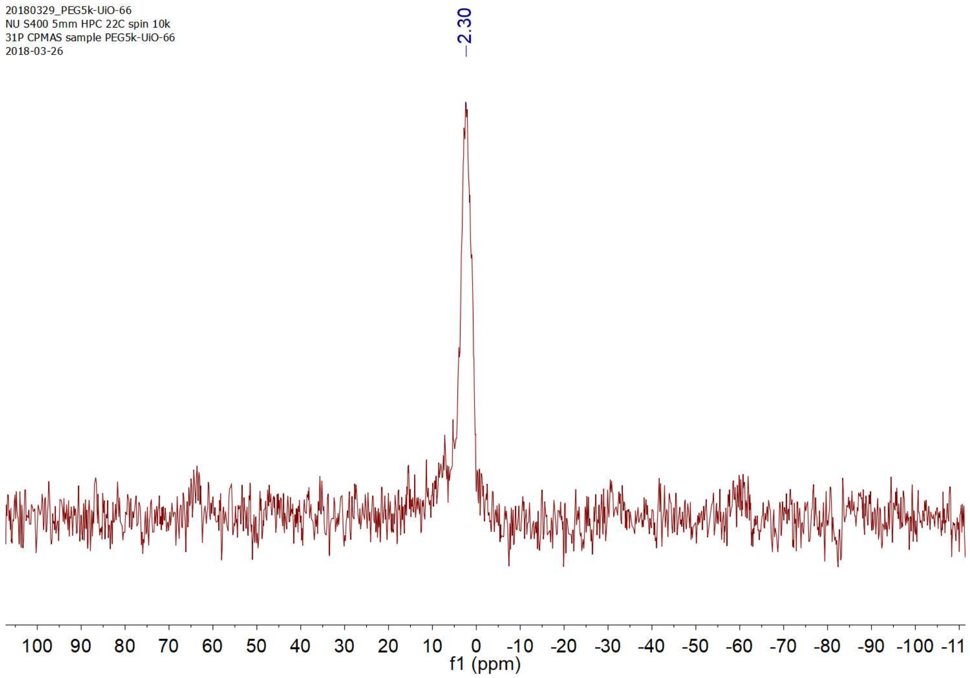

Supplementary Figure 14. $^{31}P\{^{1}H\}$ MAS NMR spectrum of phosphate-PEG_{5k}-azide ligand functionalized UiO-66 NPs.

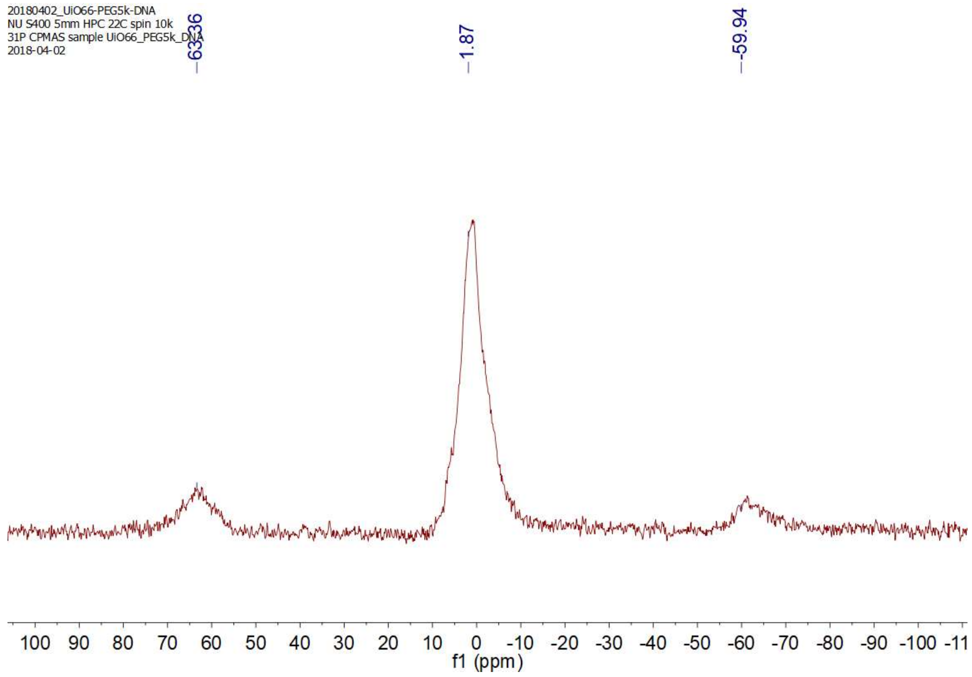

Supplementary Figure 15. $^{31}P\{^{1}H\}$ MAS NMR spectrum of DNA-PEG$_{5k}$-UiO-66 NPs.

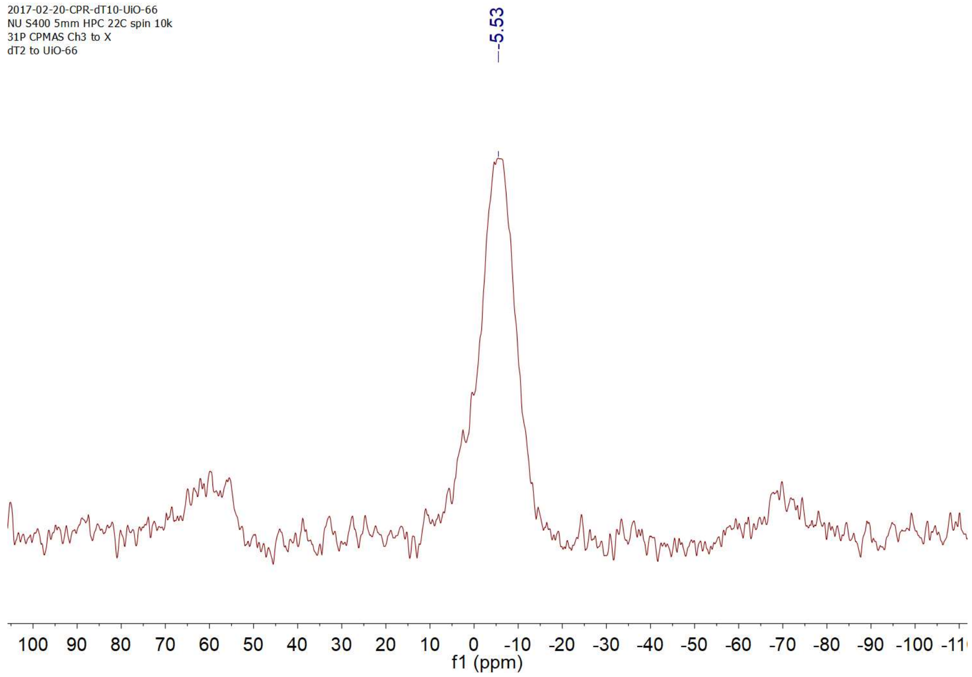

Supplementary Figure 16. $^{31}P\{^{1}H\}$ MAS NMR spectrum of unmodified DNA coordinated to the UiO-66 NPs surface via multivalent phosphate backbone bonding.

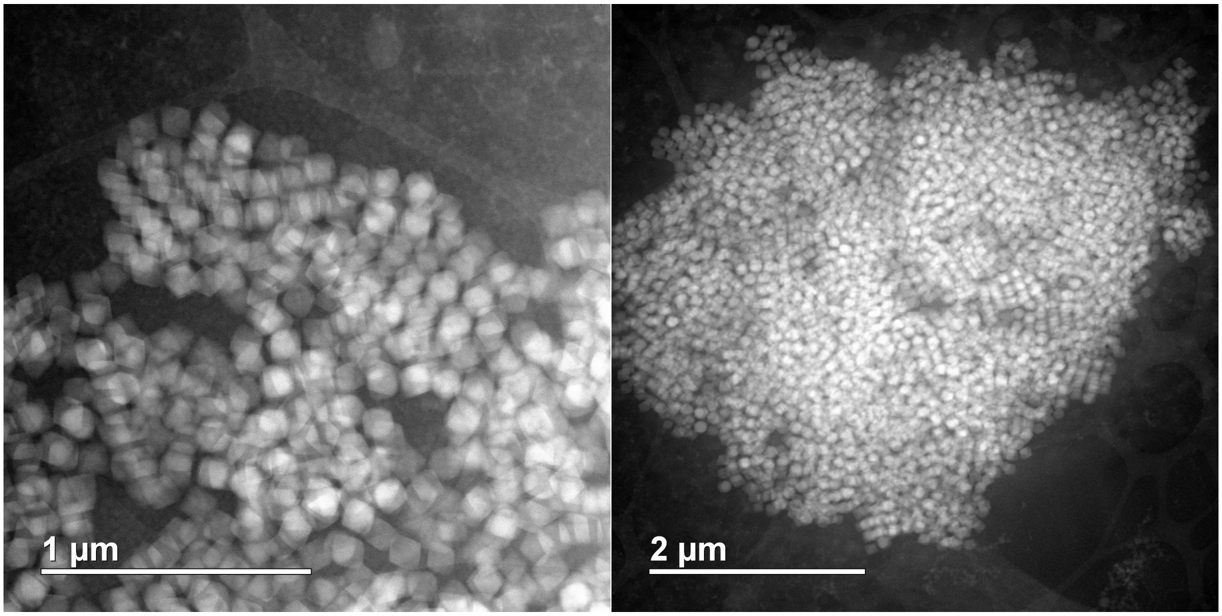

Supplementary Figure 17. Cryo-STEM micrographs of octahedral UiO-66 NP superlattices assembled with self-complementary d40 linkers.

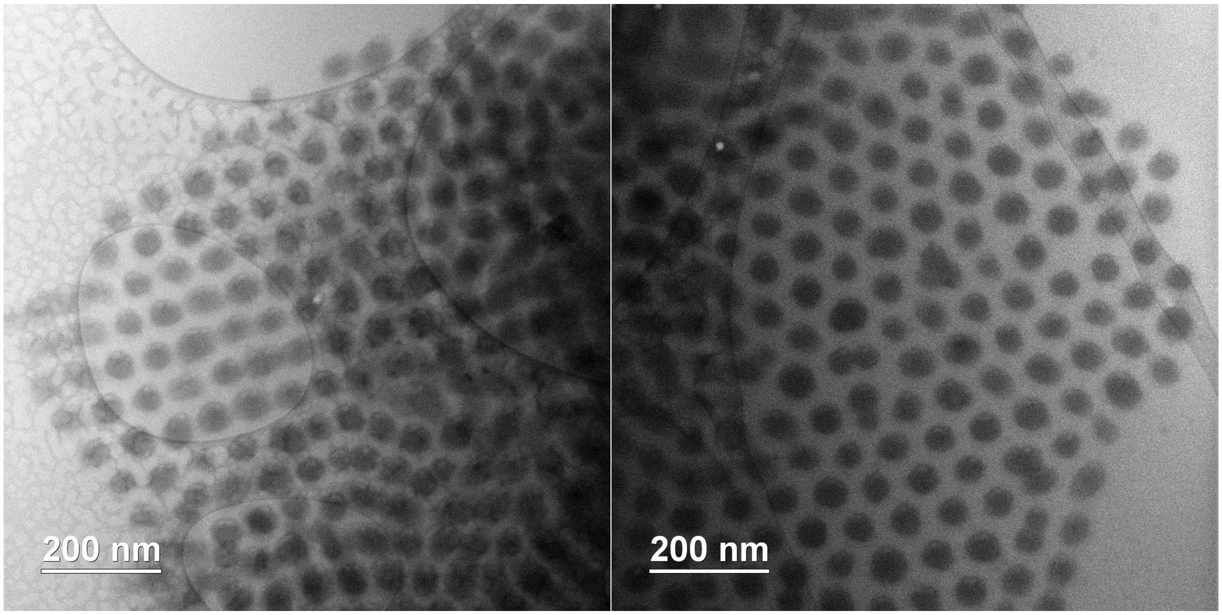

Supplementary Figure 18. Cryo-STEM micrographs of PCN-222 nanorod superlattices assembled with self-complementary d40 linkers.

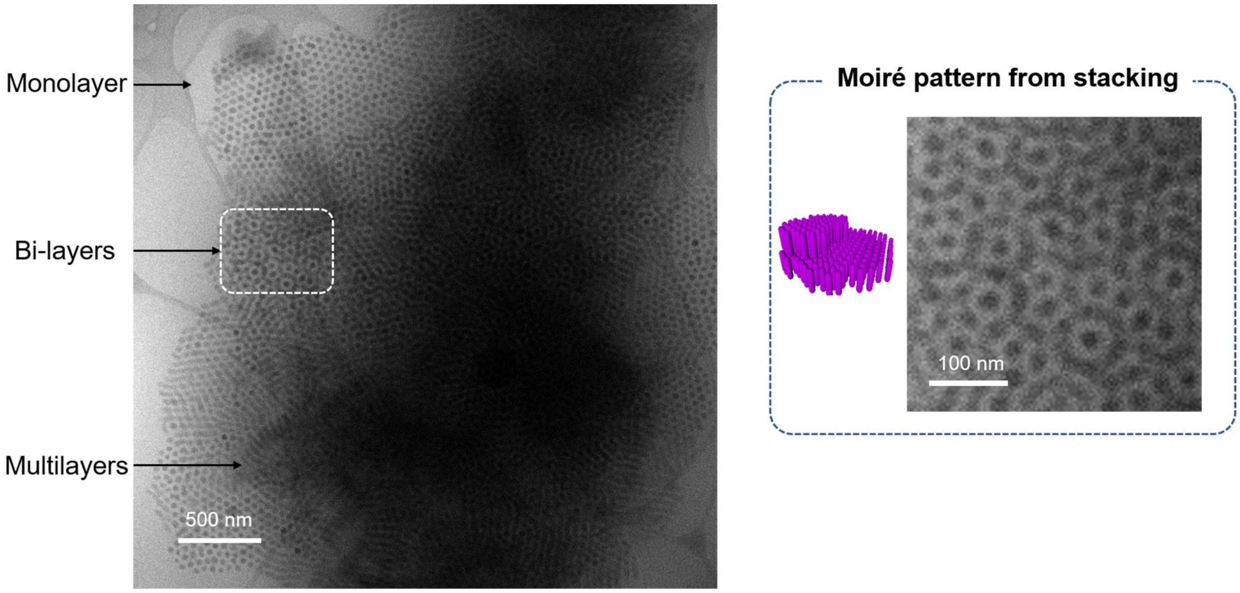

Supplementary Figure 19. Cryo-STEM micrograph of multi-layers PCN-222 nanorod assemblies showing Moire patterns (inset) from bilayer stacking.

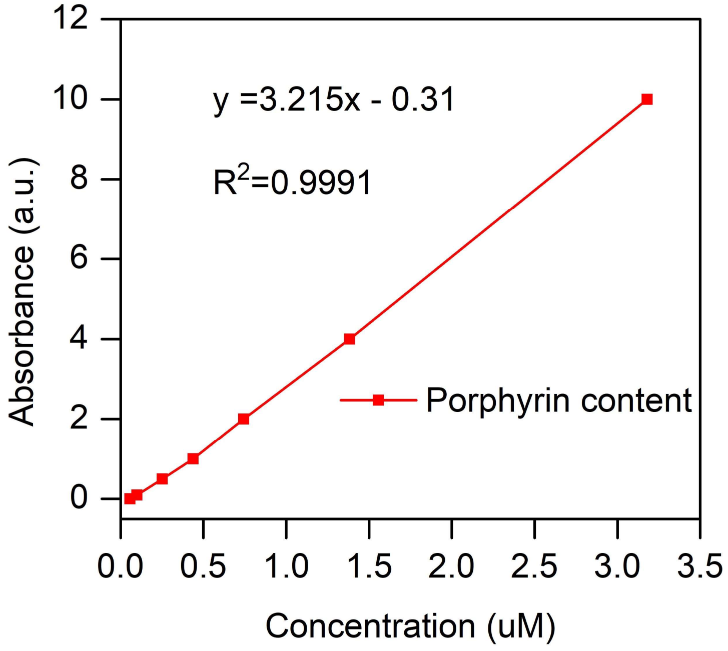

Supplementary Figure 20. UV-vis calibration curve used to quantify porphyrin concentration of the MOF samples in 0.1 M NaOH.

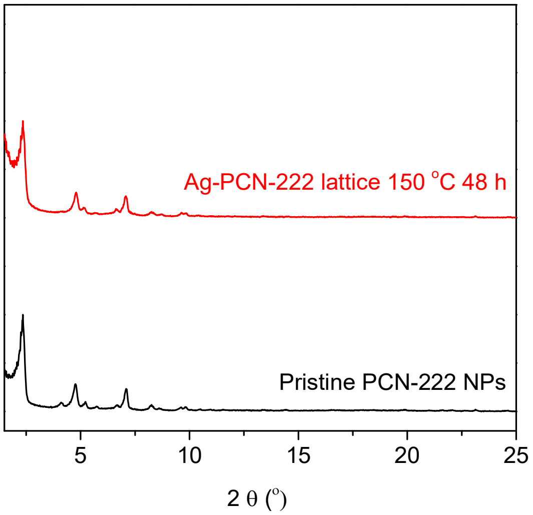

Supplementary Figure 21. PXRD patterns of pristine PCN-222 MOF NPs (black), and Ag-stabilized PCN-222 superlattice post-thermal treatment (red) at 150 °C for 48 h in the dry state.

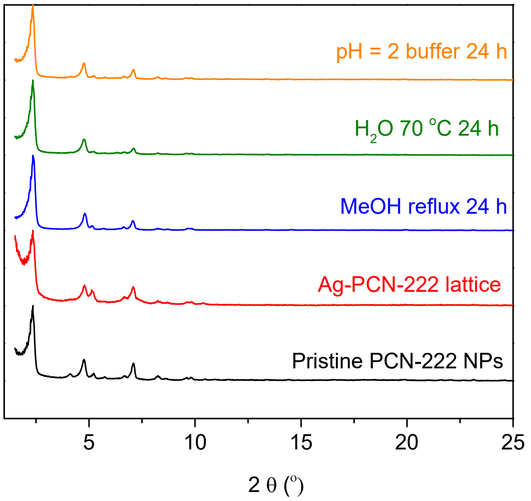

Supplementary Figure 22. PXRD patterns of the Ag-stabilized PCN-222 superlattice in the dry state (red), after refluxing in MeOH for 24 h (blue), soaking in 70 ºC water for 24 h (green), and soaking in pH = 2 buffer solution for 24 h (orange), as compared to pristine PCN-222 MOF NPs (black).

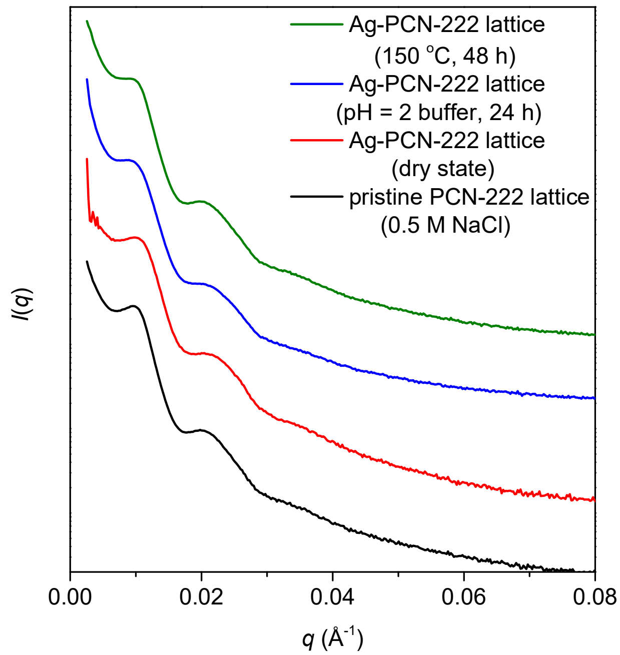

Supplementary Figure 23. SAXS patterns of the Ag-stabilized PCN-222 superlattice in the dry state (red), after soaking in pH = 2 buffer solution for 24 h (blue), and after heating to 150 °C for 48 h in the dry state (green) as compared to the as-synthesized PCN-222 superlattice dispersed in 0.5 M NaCl buffer solution (black).

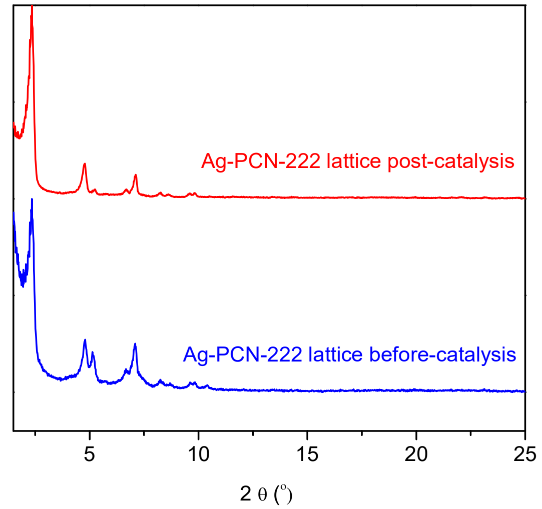

Supplementary Figure 24. PXRD patterns of Ag-PCN-222 superlattice before (blue) and after (red) 5 catalytic cycles.

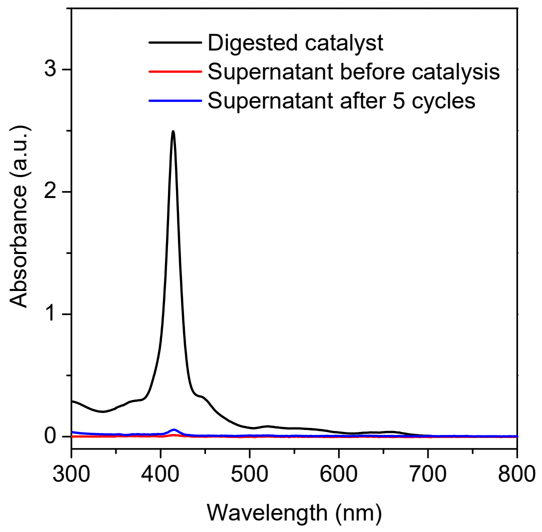

Supplementary Figure 25. Quantification of the Ag-PCN-222 superlattice catalyst leaching based on porphyrin content in the supernatant via UV-vis spectroscopy.

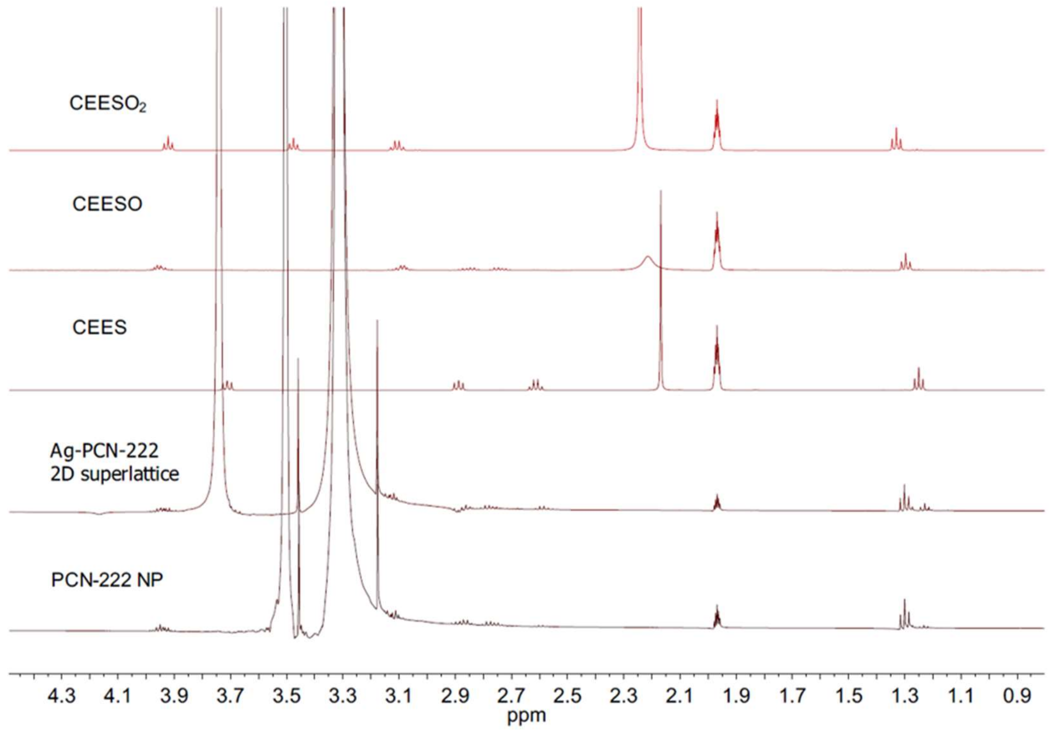

Supplementary Figure 26. NMR spectra of $CEESO_2$, CEESO, and CEES compared to the spectra for post-catalytic runs using PCN-222 2D superlattice and PCN-222 NP as catalysts. Extra peaks are assigned to methanol from the reaction mixture and residual water.

Supplementary Tables

Supplementary Table 1. Centrifugation conditions used to improve the uniformity of MOF NPs

MOF NPs	Gradient	Time (min)
**UiO-66 (37 nm)**	10%-50%	40
**UiO-66 (86 nm)**	20%-70%	40
**PCN-222 (159 nm)**	20%-70%	40

Supplementary Table 2. DNA sequences used in this study

DNA Description	DNA sequence from 5' to 3'
**AuNP-bound A**	Thiol-(PEG Spacer)$_2$-CATCCATCCTTATCAACT
**MOF-bound A**	DBCO-TEG-(PEG Spacer)$_2$-CATCCATCCTTATCAACT
**MOF-bound B**	DBCO-TEG-(PEG Spacer)$_2$-AACGACTCATACTCACCT
**Dye labelled strand**	DBCO-TEG-(PEG Spacer)$_2$-AACGACTCATACTCACCT-Tamra
**Dye labelled linker**	Tamra-TTCCTT-A-AGTTGATAAGGATGGATG
**Self-comp linker B**	CGCG-A-AGGTGAGTATGAGTCGTT
**Complementary linker A**	TTCCTT-A-(Spacer d40)$_n$-AGTTGATAAGGATGGATG
**Complementary linker B**	AAGGAA-A-(Spacer d40)$_n$-AGGTGAGTATGAGTCGTT
**Spacer d40**	TTTTTTTTTTTTT-AGTCACGACGAGTCA-TTTTTTTTTTTT-A
**Duplexer d40**	AAAAAAAAAAAA-TGACTCGTCGTGACT-AAAAAAAAAAAAA

Linker length (Spacer d40): n = 0, 1, 2, 3

All modified phosphoramidites were manufactured by Glen Research.

1. "**Thiol**" refers to the 1-O-Dimethoxytrityl-hexyl-disulfide,1'-[(2-cyanoethyl)-(N,N-diisopropyl)]-phosphoramidite (Thiol-modifier C6 S-S).

2. “**PEG Spacer**” refers to the 18-O-Dimethoxytritylhexaethyleneglycol,1-[(2-cyanoethyl)-(N,N-diisopropyl)]-phosphoramidite (Spacer phosphoramidite 18).
3. “**DBCO-TEG**” refers to 10-(6-oxo-6-(dibenzo[b,f]azacyclooct-4-yn-1-yl)-capramido-N-ethyl)-O-triethyleneglycol-1-[(2-cyanoethyl)-(N,N-diisopropyl)]-phosphoramidite (5'-DBCO-TEG phosphoramidite).
4. “**Tamra**” refers to 1-Dimethoxytrityloxy-3-[O-(N-carboxy-(Tetramethyl-rhodamine)-3-aminopropyl)]-propyl-2-O-succinoyl-long chain alkylamino-CPG (3’-Tamra CPG).

Supplementary Table 3. The C constant used to calculate interparticle distance and lattice parameters

Crystal Type	Space Group symmetry	q_0 (hkl) plane	Nearest Neighbor position	C constant
fcc	$Fm\bar{3}m$	(111)	$(\frac{1}{2}, \frac{1}{2}, 0)$	$\sqrt{6}\,\pi$
bcc	$Im\bar{3}m$	(110)	$(\frac{1}{2}, \frac{1}{2}, \frac{1}{2})$	$\sqrt{6}\,\pi$
CsCl/SC	$Pm\bar{3}m$	(100)	$(\frac{1}{2}, \frac{1}{2}, \frac{1}{2})$	$\sqrt{3}\,\pi$
AlB_2	P6/mmm	(001)	$(\frac{1}{3}, \frac{2}{3}, \frac{1}{2})$	$2\,\pi$

Supplementary References

1. Wang, S. Z. *et al.* General and Direct Method for Preparing Oligonucleotide-Functionalized Metal–Organic Framework Nanoparticles. *J. Am. Chem. Soc.* **139**, 9827-9830 (2017).
2. Kelty, M. L. *et al.* High-throughput synthesis and characterization of nanocrystalline porphyrinic zirconium metal-organic frameworks. *Chem. Commun.* **52**, 7854-7857 (2016).
3. Hurst, S. J., Lytton-Jean, A. K. R., Mirkin, C. A. Maximizing DNA loading on a range of gold nanoparticle sizes. *Anal. Chem.* **78**, 8313-8318 (2006).
4. Brunauer, S., Emmett, P. H., Teller, E. Adsorption of gases in multimolecular layers. *J. Am. Chem. Soc.* **60**, 309-319 (1938).
5. Gomez-Gualdron, D. A., Moghadam, P. Z., Hupp, J. T., Farha, O. K., Snurr, R. Q. Application of Consistency Criteria To Calculate BET Areas of Micro- And Mesoporous Metal-Organic Frameworks. *J. Am. Chem. Soc.* **138**, 215-224 (2016).
6. Li, T., Senesi, A. J., Lee, B. Small Angle X-ray Scattering for Nanoparticle Research. *Chem. Rev.* **116**, 11128-11180 (2016).
7. Auyeung, E., Macfarlane, R. J., Choi, C. H. J., Cutler, J. I., Mirkin, C. A. Transitioning DNA-Engineered Nanoparticle Superlattices from Solution to the Solid State. *Adv. Mater.* **24**, 5181-5186 (2012).
8. Macfarlane, R. J. *et al.* Nanoparticle Superlattice Engineering with DNA. *Science* **334**, 204-208 (2011).
9. Oh, T., Park, S. S., Mirkin, C. A. Stabilization of Colloidal Crystals Engineered with DNA. *Adv. Mater.* **31**, 1805480 (2019).

10. Liu, Y. Y., Howarth, A. J., Hupp, J. T., Farha, O. K. Selective Photooxidation of a Mustard-Gas Simulant Catalyzed by a Porphyrinic Metal-Organic Framework. *Angew. Chem., Int. Ed.* **54**, 9001-9005 (2015).

11. Buru, C. T. *et al.* Improving the Efficiency of Mustard Gas Simulant Detoxification by Tuning the Singlet Oxygen Quantum Yield in Metal-Organic Frameworks and Their Corresponding Thin Films. *ACS Appl. Mater. Interfaces* **10**, 23802-23806 (2018).
